# Synthesis of Ethylphosphonate Curcumin Mimics: Substituents Allow Switching Between Cytotoxic and Cytoprotective Activities

**DOI:** 10.3390/antiox14040412

**Published:** 2025-03-29

**Authors:** Valeria Romanucci, Rita Pagano, Solveigh C. Koeberle, Andreas Koeberle, Minh Bui Hoang, Sonia Di Gaetano, Domenica Capasso, Michele Francesco Maria Sciacca, Valeria Lanza, Carmelo Tempra, Fabio Lolicato, Armando Zarrelli, Danilo Milardi, Giovanni Di Fabio

**Affiliations:** 1 Department of Chemical Sciences, University of Napoli Federico II, Via Cintia 4, 80126 Naples, Italy; valeria.romanucci@unina.it (V.R.); rita.pagano@unina.it (R.P.); zarrelli@unina.it (A.Z.); 2 Institute of Pharmaceutical Sciences/Pharmacognosy and Excellence Field BioHealth, University of Graz, 8010 Graz, Austria; solveigh.koeberle@uni-graz.at (S.C.K.); andreas.koeberle@uni-graz.at (A.K.); 3 Michael Popp Institute, Center for Molecular Biosciences Innsbruck (CMBI), University of Innsbruck, 6020 Innsbruck, Austria; minh.bui-hoang@student.uibk.ac.at; 4 Institute of Biostructures and Bioimaging, National Research Council (CNR), Via P. Castellino 111, 80131 Naples, Italy; digaetan@unina.it; 5 Department of Physics Ettore Pancini, University of Naples Federico II, Via Cintia 4, 80126 Naples, Italy; domenica.capasso@unina.it; 6 Istituto di Cristallografia, National Research Council (CNR), Via Paolo Gaifami 18, 95125 Catania, Italy; michelefrancescomaria.sciacca@cnr.it (M.F.M.S.); valeria.lanza@cnr.it (V.L.); danilo.milardi@cnr.it (D.M.); 7 Institute of Organic Chemistry and Biochemistry, Czech Academy of Sciences, 160 00 Prague 6, Czech Republic; carmelo.tempra@gmail.com; 8 Heidelberg University Biochemistry Center, 69120 Heidelberg, Germany; fabio.lolicato@bzh.uni-heidelberg.de; 9 Department of Physics, University of Helsinki, 00014 Helsinki, Finland

**Keywords:** curcumin mimics, oxidative stress, apoptosis, ferroptosis, Alzheimer’s disease, cancer

## Abstract

Curcumin is recognized for its diverse biological activities, including the ability to induce apoptosis and ferroptosis. Therefore, it represents a promising candidate for the development of new compounds with neuroprotective and anticancer properties. In order to synthesize mimics with improved pharmacokinetic properties (better solubility and stability than curcumin) here, we present the design and synthesis of novel curcumin analogues named Ethylphosphonate-based curcumin mimics (EPs), which preserve the pharmacophoric features of curcumin. New EP mimics were synthesized by tyrosol- and melatonin-based building blocks using an orthogonal protection approach of the different precursors’ OH functions with good yields and in a few steps. Comparative screenings of the cytotoxic and cytoprotective properties (curcumin was used as a reference compound) were carried out on all new mimics in different cell lines (HeLa, A375, WM266, MDA-MB-231, LX2, and HDF). Assays with inhibitors of ferroptosis (Ferrostatin-1, Fer-1) and apoptosis (Quinoline-Val-Asp-difluorophenoxymethyl ketone, Q-VD), in combination with curcumin, suggested the specific cell death pathway (apoptotic or ferroptotic) of EPs, depending on the aromatic moieties contained in them. Interestingly, **EP4** exhibited substantial cytotoxic effects against various human cancer cell lines (HeLa, A375, WM266) while sparing normal cells (HDFs). **EP4** displayed a five-times-higher toxicity in triple-negative MDA-MB-231 and LX2 stellate cells than curcumin. The cytotoxicity exerted by **EP4** involves only an apoptotic mechanism, contrary to curcumin, which exerts both apoptotic and ferroptotic effects. Additionally, **EP4** was also found to be a very potent inhibitor of the ubiquitin-activating enzyme E1, reinforcing the anticancer potential of this compound. Furthermore, **EP2** possesses high antioxidant properties, efficiently protects against cell death by ferroptosis, and inhibits the amyloid aggregation involved in AD.

## 1. Introduction

**Figure 1:**
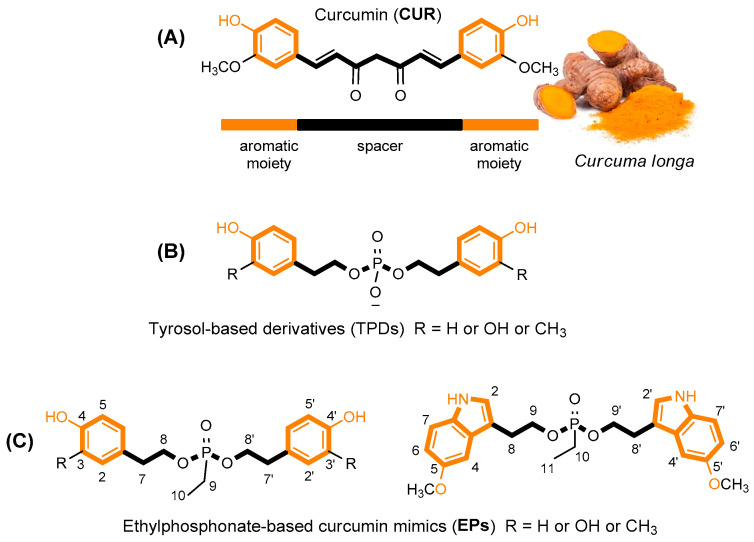
Structure of curcumin (**A**) and its tyrosol-based phosphodiester mimics (**B**) [26]. Design of new Ethylphosphonate-based curcumin mimics (**C**); this figure is adapted from the paper of Romanucci, V. et al. [26].

Natural products (NPs) derived from plants represent an invaluable source of potential therapeutic compounds. They have great potential for treating a variety of disorders, including cancer and age-related neurodegeneration. NPs exhibit vast structural diversity, are widely available, and possess a wide range of pharmacological activities [1]. What makes them even more remarkable is their capacity to perform a variety of biological functions, such as opposing reactive oxygen species, decreasing inflammation, and fighting bacteria. Nevertheless, a single molecule does not necessarily transform into a useful drug, but it could serve as a lead chemical, which would allow for the creation of new derivatives with more medicinal potential [2]. One additional interesting element is how certain NPs have demonstrated the ability to influence multiple biochemical pathways of controlled cell death, including apoptosis necroptosis and ferroptosis [3,4,5]. The complex molecular mechanisms behind ferroptosis, for example, have prompted a growing interest in the scientific community. Numerous physiological and pathological processes, including aging, organ damage, degenerative diseases, and cancer, have been linked to it [4]. Ferroptosis is an iron-dependent form of regulated cell death, characterized by toxic accumulation of lipid peroxides [6]. Therefore, researchers have identified lipophilic antioxidants and iron chelators as putative inhibitors of this cell death process since ferroptosis is linked to elevated iron levels and the accumulation of membrane-bound lipid peroxides in cells [7]. While iron accumulation and the synthesis of phospholipids containing polyunsaturated fatty acids (PUFA-PLs) are key steps in promoting ferroptosis, some control antioxidative systems, including the cyst(e)ine–glutathione (GSH) axis–glutathione peroxidase 4 (GPX4), play a crucial role in protecting against oxidative damage in ferroptosis [3]. These findings provide insight into the possibility of considering ferroptosis as a valuable therapeutic target, bringing new perspectives for the treatment of conditions like Alzheimer’s disease (AD) that are linked to oxidative stress and neuronal cell death [8,9]. Oxidative stress precedes the main neuropathologic manifestation of AD and may enhance the level of disease hallmarks, such as the accumulation of amyloid β (Aβ) peptide fibrils in senile plaques [10,11]. The development of novel ferroptosis inhibitors is currently considered as promising for treating neurodegenerative disease [3]. Furthermore, it is well known that biogenic apoptotic inducers can be considered potential anticancer agents. The ability of NPs to interfere with apoptotic and non-apoptotic cell death mechanisms provides an opportunity to exploit new molecular pathways and targets for future anticancer and/or neurodegenerative therapies [12,13]. In this respect, curcumin, a well-known polyphenol extracted from *Curcuma longa*, is one of the most studied natural products (Figure 1) [14].

It has demonstrated efficacy not only in preventing carcinogenesis but also in providing direct therapeutic benefits for various neurodegenerative disorders. Curcumin is a metabolite with multiple targets [15,16]. It plays a role in regulating ferroptosis [17,18] and inhibiting the aggregation of beta amyloid [19]. Despite its many activities, the therapeutic potential of curcumin is limited by poor pharmacokinetics, high rate of metabolism, and low stability in aqueous environments. Curcumin has recently been the focus of heated debate regarding its role as a lead compound and its therapeutic efficacy, being a compound that is practically insoluble in buffers at neutral pH [20,21]. A clear understanding of how different moieties of curcumin can influence its activity is difficult to achieve due to the complexity of the molecular diversity space of its many targets [22]. Aromatic moieties and different phenolic groups play a central role, while the size and rigidity of the spacer between the aromatic groups can vary depending on the target under consideration.

In addition, the methylene group and β-diketone moiety contribute to its instability, poor absorption, and rapid metabolism. In view of the ongoing active debate in the medicinal chemistry community regarding the therapeutic efficacy of the parent curcumin, an ongoing search for curcumin-inspired compounds that could overcome these challenges in drug discovery seems noteworthy. So, research is still active today in the design of synthetic analogues aimed at replacing the central diketone structure with a moiety that enhances chemical stability, water solubility, and bioavailability while preserving the 3-methoxy-tyrosol moiety [23,24]. Different approaches have been explored to link two aromatic moieties through spacers with different rigidities to create libraries of new curcumin mimics with high structural diversity and pharmacological activity. The aromatic groups, their distance, and the presence of phenolic moieties have been variable elements used to outline the structure–activity relationships of the new mimics [25].

Recently, we proposed the synthesis of tyrosol phosphodiester derivatives (TPDs) as curcumin mimics, structures containing building blocks known for their pharmacological activities, such as tyrosol (TYR), hydroxytyrosol (HTYR), and 3-methoxytyrosol (MTYR), linked through a phosphodiester bridge (Figure 1) [26,27]. Structurally, TPDs retain the two aromatic rings with different hydroxyl substituents, and the distance between them is comparable to that of curcumin. The new compounds have demonstrated interesting anti-fibrillogenic and anticancer activities, and the crucial role of the catechol (4-hydroxyphenol) and guaiacol (4-hydroxy-3-methoxyphenol) units of HTYR and MTYR, respectively, have been highlighted. Continuing these studies on tyrosol-based analogues, here we report the design and synthesis of curcumin analogues containing an ethylphosphonate group in the linker (EP, Figure 1). We applied this molecular modification to improve the pharmacological profile of bioactive curcumin, while preserving the relevant pharmacophoric features of the lead structure also found in TPDs: the aromatic groups and the phenolic functions.

The EP design focuses on removing a charge on the phosphate group and replacing it with a neutral ethyl phosphonate one, which has been applied to improve the delivery of new compounds. Mainly, the modification was made to the spacer structure, which was designed to be more stable than that of curcumin. The distance between the aromatic moieties was almost unchanged. In addition to tyrosols, we also included a melatonin-like scaffold as a promising building block for its effective free radical scavenging, anti-inflammatory properties, and good lipophilicity. The solubility, stability, and antioxidant activity were preliminarily investigated, together with the prediction of the ability of these mimics to permeate the cell membrane. New mimics were screened in vitro on different cancer cell lines; the ferroptotic and apoptotic mechanisms involved in the cell death process were investigated. Interesting results emerged from studies on specific targets involved in neurodegeneration and carcinogenesis, such as amyloid peptide aggregation and the activation of ubiquitin-mediated signaling.

## 2. Materials and Methods

### 2.1. General

All chemicals were purchased from Sigma-Aldrich (Milano, Italy). HPLC-grade ACN and MeOH were purchased from Carlo Erba Reagents (Milano, Italy) and Sigma-Aldrich, respectively. Reactions were monitored by TLC (F254-precoated silica gel plates, Merck) and column chromatography (Merck Kieselgel 60, 70–230 mesh, Milano, Italy). HPLC analysis was performed with a Shimadzu LC-8A HPLC system (Shimadzu Analytical and Measuring Instruments, Milano, Italy) equipped with a Shimadzu SCL-10A VP System control and a Shimadzu SPD-10A VP UV-Vis detector. Mass spectrometric analyses were performed on the AB SCIEX TOF/TOF 5800 in positive or negative mode and the Waters Micromass ZQ Instrument (Waters, Milano, Italy) equipped with an electrospray source in positive mode. The NMR spectra were recorded at 25 °C on an NMR spectrometer, Bruker DRX, Bruker Advance (Bruker Italia Srl, Milano, Italy), and the INOVA-500 NMR instrument (Varian, Milan, Italy). Ubiquitin-activation enzyme (UBE1) and UbcH13/Uev heterodimer complex were obtained from Boston Biochem. Amyloid β peptide 1–40 (Aβ) of a purity greater than 95% was provided by GenScript. Thioflavin T (ThT) was obtained from Sigma-Aldrich, while Hexafluoroisopropanol (HFIP) was sourced from Carlo Erba.

### 2.2. Synthesis of Ethylphosphonate-Linked Tyrosol Dimers (**EP1**–**EP4**): General Coupling Procedure

A total of 1 mmol of compounds **1**–**4** was dissolved in 1.5 mL of dry DCM in the presence of 2 mmol of TEA at 0 °C. Subsequentially, 0.5 mmol of ethyl dichlorophosphate was added, and the reaction was stirred at rt for 2 h, monitoring by TLC (hexane/EtOAc 60:40, *v*/*v*). The reaction was quenched with H_2_O and extracted using H_2_O/DCM. The combined organic phases were dried over anhydrous Na_2_SO_4_, evaporated under reduced pressure, and then coarse-purified by column chromatography eluted with EtOAc/hexane 80:20 (*v*/*v*).

A total of 0.24 mmol of protected compounds was then dissolved in 1 mL of THF and reacted with the complex TEA·3HF (2.5 equiv. for each TBDMS group) at rt for 2 h. The reaction mixture was dried under reduced pressure and purified by column chromatography eluted with DCM/MeOH 95:5 (*v*/*v*), obtaining the final compounds **EP1**–**EP4** with yields of 34–75%. All compounds, **EP1**–**EP4**, were purified by RP-HPLC using a Phenomenex Gemini RP18 column (10-μm particle size, 21.20 mm × 250 mm i.d.) with a linear gradient of ACN in H_2_O, from 20% to 100% over 30 min at a flow rate of 7 mL/min with detection at 280 and 260 nm. The identity of compounds **EP1**–**EP4**, with a final purity ≥ 99%, were confirmed by 1D and 2D NMR and MALDI-TOF analyses.

**EP1**  R*_f_* = 0.6 (DCM-MeOH 9:1, *v*/*v*). HPLC purity ≥ 99%; ^1^H NMR (CD_3_OD, 400 MHz, rt): δ = 7.04 (4H, *d*, *J_2_*_,*3*_ *= J_2′_*_,*3′*_ = 8.31 Hz, H-3, H-3′, H-5, H-5′); 6.73 (4H, *d*, *J_2_*_,*3*_ *= J_2′_*_,*3′*_ = 8.31 Hz, H-2, H-2′, H-6, H-6′); 4.06 (4H, complex signal, H-8, H-8′); 2.82 (4H, *t*, *J_7_*_,*8*_ *= J_7′_*_,*8*_ = 6.79 Hz, H-7, H-7′); 1.64 (2H, *dq*, *J_9a__–__9b_ =* 7.7 Hz, *J_9-P_ =* 18.1 Hz, H-9); 1.01 (3H, *dt*, *J_9–10_ =* 7.6 Hz, *J_10-P_ =* 20.3 Hz, H-10) ppm. ^13^C NMR (CD_3_OD, 100 MHz, rt): δ = 155.9 (2C, C-4, C-4′); 129.7 (4CH, C-3, C-3′, C-5, C-5′); 128.1 (2C, C-1, C-1′); 114.9 (4CH, C-2, C-2′, C-6, C-6′); 66.5 (*d*, *^2^J_C8-P_* = *^2^J_C8′-P_* = 7.35 Hz, 2CH_2_, C-8, C-8′); 35.6 (*d*, *^3^J_C7-P_* = *^3^J_C7′-P_* = 6.32 Hz, 2CH_2_, C-7, C-7′); 17.4 (*d*, *J_C9-P_* = 142.6 Hz, CH_2_, C-9); 5.10 (*d*, *^2^J_C10-P_* = 6.64 Hz, CH_3_, C-10) ppm. ^31^P NMR (CD_3_OD, 161.98 MHz, rt): δ = 34.7 ppm. HRMS (MALDI-TOF, positive ions): m/z calculated for C_18_H_24_O_5_P^+^ = 351.1356; found: 351.1408 [M+H]^+^.
**EP2**  R*_f_* = 0.5 (DCM-MeOH 9:1, *v*/*v*). HPLC purity ≥ 99%; ^1^H NMR (CD_3_OD, 400 MHz, rt): δ = 6.70 (2H, *d*, *J_5_*_,*6*_ *= J_5′_*_,*6′*_ = 8.1 Hz, H-5, H-5′); 6.67 (2H, *d*, *J_2_*_,*5*_ *= J_2′_*_,*5′*_ = 1.8 Hz, H-2, H-2′); 6.54 (2H, *dd*, *J_6_*_,*5*_ *= J_6′_*_,*5′*_ = 8.0, *J_6_*_,*3*_ *= J_6′_*_,*3′*_ = 1.6 Hz, H-6, H-6′); 4.06 (4H, complex signal, H-8, H-8′); 2.77 (4H, *t*, *J_7_*_,*8*_ *= J_7′_*_,*8′*_ = 6.6 Hz, H-7, H-7′); 1.64 (2H, *dq*, *J_9a_*_,*9b*_ = 7.6 Hz, *^2^J_9-P_ =* 18.1 Hz, H-9); 1.02 (3H, *dt*, *J_9_*_,*10*_ = 7.8 Hz *^3^J_10-P_ =* 20.2 Hz, H-10) ppm. ^13^C NMR (CD_3_OD, 100 MHz, rt): δ = 144.8 (2C, C-4, C-4′); 143.6 (2C, C-3, C-3′); 128.9 (2C, C-1, C-1′); 120.0 (2CH, C-6, C-6′); 115.8 (2CH, C-2, C-2′); 115.0 (2CH, C-2, C-2′); 66.5 (*d*, *^2^J_C8-P_* =*^2^J_C8′-P_* = 6.90 Hz, 2CH_2_, C-8, C-8′); 35.9 (*d*, *^3^J_C7-P_* = *^3^J_C7′-P_* = 6.6 Hz, 2CH_2_, C-7, C-7′); 17.4 (*d*, *J_C9-P_* = 141.8 Hz, CH_2_, C-9); 5.1 (*d*, *^2^J_C10-P_* = 6.5 Hz, CH_3_, C-10) ppm. ^31^P NMR (CD_3_OD, 161.98 MHz, rt): δ = 34.7 ppm. HRMS (MALDI-TOF, negative ions): *m*/*z* calculated for C_18_H_22_O_7_P^−^ = 381.1109; found: 381.1567 [M+H]^−^.
**EP3**  R*_f_* = 0.5 (DCM-MeOH 9:1, *v*/*v*). HPLC purity ≥ 99%; ^1^H NMR (CD_3_OD, 400 MHz, rt): δ = 6.81 (2H, *d*, *J_2_*_,*5*_ *= J_2′_*_,*5′*_ = 1.5 Hz, H-2, H-2′); 6.74 (2H, *d*, *J_5_*_,*6*_ *= J_5′_*_,*6′*_ = 8.1 Hz, H-5, H-5′); 6.65 (2H, *dd*, *J_6_*_,*5*_ *= J_6′_*_,*5′*_ = 8.1, *J_6_*_,*3*_ *= J_6′_*_,*3′*_ = 1.67 Hz, H-6, H-6′); 4.09 (4H, complex signal, H-8, H-8′); 3.83 (6H, *s*, -OCH_3_); 2.83 (4H, *t*, *J_7_*_,*8*_ *= J_7′_*_,*8′*_ = 6.6 Hz, H-7, H-7′); 1.65 (2H, *dq*, *J_9a-9b_ =* 7.6 Hz, *^2^J_9-P_ =* 18.0 Hz, H-9); 1.02 (3H, *dt*, *J_9–10_ =* 7.6 Hz, *^3^J_10-P_ =* 20.1 Hz, H-10) ppm. ^13^C NMR (CD_3_OD, 100 MHz, rt): δ = 147.5 (2C, C-3, C-3′); 144.9 (2C, C-4, C-4′); 128.9 (2C, C-1, C-1′); 121.2 (2CH, C-6, C-6′); 114.8 (2CH, C-5, C-5′); 112.3 (2C, C-2, C-2′); 66.5 (*d*, *^2^J_C8-P_* = *^2^J_C8′-P_* = 6.9 Hz, 2CH_2_, C-8, C-8′); 55.0 (CH_3_, -OCH_3_); 36.0 (*d*, *^3^J_C7-P_* = *^2^J_C7′-P_* = 6.2 Hz, 2CH_2_, C-7, C-7′); 17.5 (*d*, *J_C9-P_* = 141.9 Hz, CH_2_, C-9); 5.2 (*d*, *^2^J_C10-P_* = 6.9 Hz, CH_3_, C-10) ppm. ^31^P NMR (CD_3_OD, 161.98 MHz, rt): δ = 34.7 ppm. HRMS (MALDI-TOF, positive ions): m/z calculated for C_20_H_28_O_7_P^+^ = 411.1567; found: 411.1593 [M+H]^+^.
**EP4**  R*_f_* = 0.6 (DCM-MeOH 9:1, *v*/*v*). HPLC purity ≥ 99%; ^1^H NMR (DMSO-d6, 500 MHz, rt): δ = 10.7 (2H, *s*, NH); 7.23 (2H, *d*, *J_6_*_,*7*_ *= J_6′_*_,*7′*_ = 8.8 Hz, H-7, H-7′); 7.14 (2H, *s*, H-2, H-2′); 7.02 (2H, *d*, *J_4_*_,*6*_ *= J_4′_*_,*6′*_ = 1.5 Hz, H-4, H-4′); 6.72 (2H, *dd*, *J_6_*_,*7*_ *= J_6′_*_,*7′*_ = 8.6, *J_6_*_,*4*_ *= J_6′_*_,*4′*_ = 2.0 Hz, H-6, H-6′); 4.13 (4H, complex signal, H-9, H-9′); 3.74 (6H, *s*, -OCH_3_); 2.99 (4H, *t*, *J_8_*_,*9*_ *= J_8′_*_,*9′*_ = 7.0 Hz, H-8, H-8′); 1.67 (2H, *dq*, *J_10–11_ =* 7.7 Hz, *^2^J_10-P_ =* 18.0 Hz, H-10); 0.96 (3H, *dt*, *J_10–11_ =* 7.7 Hz, *^3^J_11-P_ =* 19.8 Hz, H-11) ppm. ^13^C NMR (DMSO-d6, 100 MHz, rt): δ = 153.5 (2C, C-5, C-5′); 131.7 (2C, C-7a, C-7a’); 127.9 (2C, C-4a, C-4a’); 124.3 (2CH, C-2, C-2′); 112.5 (2CH, C-7, C-7′); 111.6 (2CH, C-6, C-6′); 110.0 (2C, C-3, C-3′); 100.5 (2CH, C-4, C-4′); 65.4 (*d*, *^2^J_C9-P_* = 6.7 Hz, 2CH_2_, C-9, C-9′); 55.8 (CH_3_, -OCH_3_); 26.9 (*d*, *^3^J_C8-P_* = 5.6 Hz, 2CH_2_, C-8, C-8′); 18.2 (*d*, *J_10-P_* = 141.09 Hz, CH_2_, C-10); 6.8 (*d*, *^2^J_C11-P_* = 6.61 Hz, CH_3_, C-11) ppm. ^31^P NMR (DMSO-d6, 161.98 MHz, rt): δ =33.4 ppm. HRMS (MALDI-TOF, positive ions): m/z calculated for C_24_H_30_N_2_O_5_P^+^ = 457.1887; found: 457.1916 [M+H]^+^.

### 2.3. Cell Culture of Human Cancer Cells (HeLa, A375, WM266, MDA-MB-231, LX2) and HDF Cell Lines

Human adenocarcinoma (HeLa), human melanoma (A375), and human normal dermal fibroblasts (HDFs) were grown in DMEM with 10% fetal bovine serum (FBS), 1% glutamine, 100 U/mL penicillin, and 100 μg/mL streptomycin (Euroclone, Italy). The human metastatic melanoma cell line (WM266) was grown in RPMI with 10% fetal bovine serum (FBS), 1% glutamine, 100 U/mL penicillin, and 100 μg/mL streptomycin. The cells were maintained in humidified air containing 5% CO_2_ at 37 °C. Human triple-negative MDA-MB-231 breast cancer cells were obtained from the American Type Culture Collection (ATCC, Manassas, VA, USA). MDA-MB-231 cells (2 × 10^4^ cells/cm^2^) and LX2 cells (1.6 × 10^4^ cells/cm^2^) were cultured in DMEM (glucose 4.5 g/L, Thermo Fisher Scientific, Waltham, MA, USA) supplemented with 10% heat-inactivated fetal calf serum (Merck) and, in the case of MDA-MB-231 cells, additionally 100 U/mL penicillin and 100 g/mL streptomycin (Thermo Fisher Scientific) at 37 °C in a 5% CO_2_ atmosphere. Cells were routinely tested for mycoplasma contamination using the MycoAlert™ PLUS Mycoplasma Detection Kit from Lonza (Basel, Switzerland). In addition, their morphology was examined regularly.

### 2.4. Determination of Metabolic Activity

For the cell proliferation assay, HeLa and A375 cells were seeded at a density of 1200 cells/100 μL and WM266 and HDFs at a density of 2000 cells/100 μL on 96-well plates. Each molecule (10 or 50 μM), or DMSO as vehicle, was added to the cells. After 48 h of incubation at 37 °C, cell viability was assessed by performing the 3-(4,5-dimethylthiazol-2-yl)-2,5- diphenyltetrazolium bromide (MTT, Sigma Aldrich, Italy) reduction inhibition assay. Cytotoxicity experiments were independently performed at least three times. Cell viability was expressed as a percentage with respect to the control represented by cells treated with vehicle alone. Statistical significance was obtained by Student’s *t*-test paired, two-sided. Data are expressed as means standard error (S.E.). *p* values < 0.05 were statistically significant. The IC50 values were obtained by the Prism 6.01 software (GraphPad San Diego, CA, USA) by extrapolating them from the dose–response curve data.

MDA-MB-231 and LX2 cells were seeded in triplicate in 96-well plates at a density of 20,000 cells per well in 100 μL of medium and allowed to attach for 24 h at 37 °C and 5% CO_2_. Compounds were added at a final DMSO concentration of 0.5%, and cells were incubated for an additional 48 h. The pan-kinase inhibitor staurosporine (1 µM, Merck) was used as a reference compound, and the concentration was selected to efficiently induce cell death (≥90%). To identify the cell death program, the apoptosis inhibitor Q-VD-OPh (50 µM, Merck) and the ferroptosis inhibitor ferrostatin-1 (10 µM, Cayman Chemicals, Ann Arbor, MI) were added to the medium 2 h before treatment with the cytotoxic compounds. The concentrations of Q-VD-OPh and ferrostatin-1 were chosen based on commonly applied ranges reported in the literature [28,29] and were further optimized in preliminary experiments using the apoptosis inducers etoposide and staurosporine, as well as the ferroptosis inducer RSL3, to ensure suppression of apoptosis and ferroptosis, respectively. The final vehicle (DMSO) concentration was adjusted to 0.5% (IC_50_ values) and 1% (inhibitor studies). For the MTT assay, 20 µL of 3-(4,5-dimethylthiazol-2-yl)-2,5-diphenyltetrazolium bromide (MTT, 5 mg/mL in PBS pH = 7.4, sterile-filtered, Merck) solution was added to each well, and the cells were incubated for 1.5 h. Then, 100 μL of SDS solution (10% in 20 mM HCl, pH = 4.5) was added, and the plates were shaken at 130 rpm in the dark for 18–20 h. Absorbance was measured at 570 nm using a SpectraMax iD3 plate reader (Molecular Devices). Absorbance values from ethanol-treated cells were used for background subtraction, and cell viability was expressed as percentage relative to the vehicle control (DMSO). An asymmetric sigmoidal (5 PL) curve fit was performed using GraphPad Prism 9.

### 2.5. Wound Healing Assay

In vitro cell migration was evaluated using the wound healing scratch assay. A total of 600,000 WM266 cells were plated and grown to confluence. Successively, the cells were linearly scratched with a pipette tip to generate the wound. Once the detached cells were removed, the molecule was added at the indicated concentrations and the cells were incubated at 37 °C. Afterwards, the scratch area was photographed at 0, 24, and 48 h [30] using a phase optical microscope at 10× magnification (Zeiss) and the distance between the edges of the incisions was measured. The mean value was determined as follows: wound closure (%) = 1× (wound width tx/wound width t_0_) ×100. Bars depict the mean ± SE of three independent experiments.

### 2.6. Inhibition of RSL3-Induced Ferroptotic Cell Death

The anti-ferroptotic properties of the compounds were evaluated in MDA-MB-231 cells. Cells were seeded in 96-well plates at a density of 20,000 cells per well in 100 μL of medium. After 24 h at 37 °C and 5% CO_2_, cells were treated with the vehicle (0.5% DMSO) or the compounds at 3 µM and 30 µM alone or in combination with RSL3 (0.3 μM, Cayman Chemicals) for an additional 48 h. The cellular dehydrogenase activity was assessed by the reduction of MTT, as described in Section 2.4.

### 2.7. Ubiquitin Activation Assays

During the Ub activation process, the E1 enzyme binds ATP–Mg^2+^ and Ub, catalyzing the acyl adenylation of the Ub C-terminus while releasing pyrophosphate. Subsequently, an E1 cysteine attacks the Ub–AMP complex via acyl substitution, forming a high-energy Ub–E1 thioester bond. Pyrophosphate release was monitored through a colorimetric assay at λ = 790 nm, using the 96-well plate reader Varioskan (ThermoFisher, Waltham, MA, USA) with a 5 min time interval. Polyubiquitination reactions were conducted in the presence of EPi or curcumin (concentration range 0–5 μM) in 10 μL of activation buffer (50 mM Tris–HCl, 30 mM MgCl_2_, 13 μM DTT, and 2.6 mM ATP) containing Ub (80 μM), E1 (100 nM), and E2 (300 nM) at 37 °C for 1 h. Quenching of all reactions was achieved by adding 10 μL of 400 mM EDTA solution per well, followed by incubation with 200 μL of 3 mM ammonium heptamolybdate in 0.6 M HCl (60% acetonitrile/water) for 10 min. The colorimetric reaction was initiated by adding 80 μL of ascorbic acid (500 mM) in 2 M HCl (60% ACN/W) and promptly analyzed. Results are presented as the percentage of E1 activity compared to the control, with analysis of variance conducted using one-way ANOVA. To determine the IC_50_ value of EP4, the concentration that reduces the value of E1 activation by half, the normalized responses as a function of the concentrations of the **EP4** were analyzed using the following equation:

$$I=I_{0}+\frac{I_{max}-I_{0}}{1+\frac{\left[x\right]}{{IC}_{50}}}$$

where *I*_0_ and *I_max_* are the minimum and maximum values of inhibition and [x] is the concentration of **EP4**.

### 2.8. ThT Assays

Initially, Aβ peptides were dissolved in HFIP at a concentration of 1 mg/mL. These solutions were then divided into 100 μL aliquots, frozen at –80 °C, and subjected to lyophilization. Quantification was performed by reconstituting aliquots in 20 μL of 1 mM NaOH, followed by addition to a 180 μL buffer solution (10 mM phosphate, pH = 7.4), with absorbance at 280 nm measured for quantification using an extinction coefficient (ε = 280 nm) of 1450 M^−1^ cm^−1^. For ThT assays, samples were prepared by adding 1 μL of each ligand stock solution in water (final concentrations of 5 μM) to 50 μL of ThT solution in PBS (10 mM phosphate, pH = 7.4, and 100 mM NaCl, at a concentration of 20 μM), in 384-well plates. Following the addition of Aβ (final concentration of 10 μM), time traces were recorded using a Varioskan plate reader (ThermoFisher, Waltham, MA, USA) with excitation at 440 nm (λ_ex_) and emission at 480 nm (λ_em_). The readings were taken at 37 °C with the plate shaken at 600 rpm and a 1 mm orbital movement diameter for 10 s before each reading.

## 3. Results and Discussion

### 3.1. Synthesis and Characterization of EPs

**Scheme 1:**
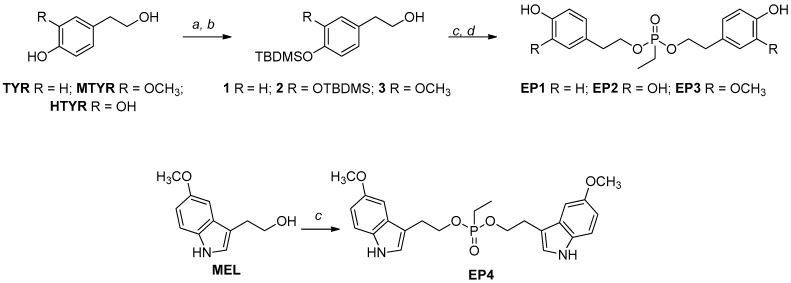
Synthetic routes of EP curcumin mimics. Reagents and conditions: (*a*) TBDMSCl, Et_3_N, DMF, r.t., 3 h; (*b*) 1% I_2_/MeOH (*w*/*v*), r.t.; (*c*) ethylphosphonic dichloride, DCM, TEA, 0 °C, 2 h; (*d*) Et_3_N∙3HF in THF, rt, 2 h.

For the synthesis of EPs, we started with tyrosols identified as tyrosol (TYR), 3-methoxy-tyrosol (MTYR), and 3-hydroxy-tyrosol (HTYR), which differ in the substituent on the aromatic ring, and from 5-methoxytryptophol (MEL), which possess an indole ring (Scheme 1).

Where necessary, the TBDMS group was chosen to protect the phenolic functionalities due to the easy installation procedure and the mild regioselective removal conditions with I_2_ in MeOH to obtain the building blocks with free primary hydroxyl function [26] with a good yield (82–86%). These scaffolds (**1**–**3**) were reacted with a bidentate phosphorylating agent, and after the treatment with the complex TEA∙3HF in THF, to remove TBDMS groups, the new mimics were obtained with about 48–75% overall yields (**EP1**–**EP3**).

In the case of MEL, the reaction with the phosphorylating agent was carried out directly, and the new mimic, **EP4**, was obtained with about a 34% overall yield. The lower yield, compared to the others, was due to the presence of the indole nitrogen, which, as a nucleophile, can give unwanted products. The reaction was optimized in different experimental conditions, such as temperature and stoichiometric ratios. All new compounds were purified by RP-HPLC and fully characterized by ^1^H, ^13^C, and ^31^P NMR. The NMR and MS analyses confirmed the formation of the expected products.

From the NMR spectra analysis, it was possible to highlight the diasterotopic and enantiotopic nature of the H-8 (or H-9 in **EP4**, Figure 1) and -CH_2_ bound to the phosphorus nucleus, respectively. In all cases, the H-8 and H-8′ (or H-9 and H-9′ in **EP4**) protons appeared, in the ^1^H NMR spectrum, as complex overlapped signals (see Supplementary Materials).

Due to the presence of a phosphorus nucleus, the protons of the ethyl group appear as A_2_M_3_X systems. In detail, the −CH_2_ (A_2_) protons are enantiotopic, and they appear as a double quartet (*J*_H-H_ = 7.7 Hz, *J*_H-P_ = 18.1 Hz), and CH_3_ (M_3_) appears as a double triplet (*J*_H-H_ = 7.6 Hz, *J*_H-P_ = 20.3 Hz). In the ^13^C NMR spectra, the couplings with the phosphorus nucleus of C8 (or C9 in **EP4**, Figure 1), as well as those of the ethyl group, are also observed (see Supplementary Materials).

### 3.2. Chemical Stability, Water Solubility, and Radical Scavenger Activities of EPs

The stability of **EP1** as a representative compound was investigated to ensure that the observed effects arise from the compounds themselves and not from by-products or the mixture of components. The percentage of **EP1** remaining over time was followed by HPLC experiments performed under different buffer conditions and pHs. Its stability was evaluated over time at 37 °C in PBS pH = 7.4 and simulated gastric fluid (sGF, pH = 1.2) for up to 24 h (for details, see Supplementary Materials). The time-dependent stability in phosphate buffer at pH = 7.4 did not markedly differ from that observed in sGF (Figure S1 in Supporting Information). Substantial amounts of **EP1** were present even after 48 h (≥93%). The water solubility of the new mimics was assessed in PBS at pH 7.4 by measuring UV absorbance at 280 nm across various concentrations (for details, see Supplementary Materials). Amounts were determined via UV–absorption calibration curves (R^2^ = 0.999).

All compounds exhibited higher solubility compared to that reported in several papers for curcumin (insoluble in water at room temperature and neutral pH) [31,32]. **EP2** and **EP3** were the most soluble, with a concentration of 40 µM for both compounds, while **EP1** and **EP4** demonstrated solubilities of 15 µM and 20 µM, respectively (Table 1). The antioxidant activity of EPs was assessed using 2,2-diphenyl-1-picrylhydrazyl (DPPH) and oxygen radical absorbance capacity (ORAC) assays, which measure free radical scavenging and peroxyl radical neutralization, respectively. The antioxidant activities are presented in Table 1 together with the four starting compounds, TYR, MTYR, HTYR, and MEL, and curcumin as a reference (for details, see Supplementary Materials). All new mimics (**EP1**–**EP4**) showed a greater antioxidant activity in the ORAC assay than the starting compounds (Table 1), while in the DPPH assay, only **EP2** and **EP3** showed significant antioxidant activity. The compound **EP2** displayed the highest antioxidant power, exceeding that of curcumin in both assays. As expected, the catechol moiety appears to be essential for antioxidant activity.

### 3.3. Free Energy Profiles of **EP1**–**EP4** Curcumin-Inspired Compounds During Membrane Translocation

**Figure 2:**
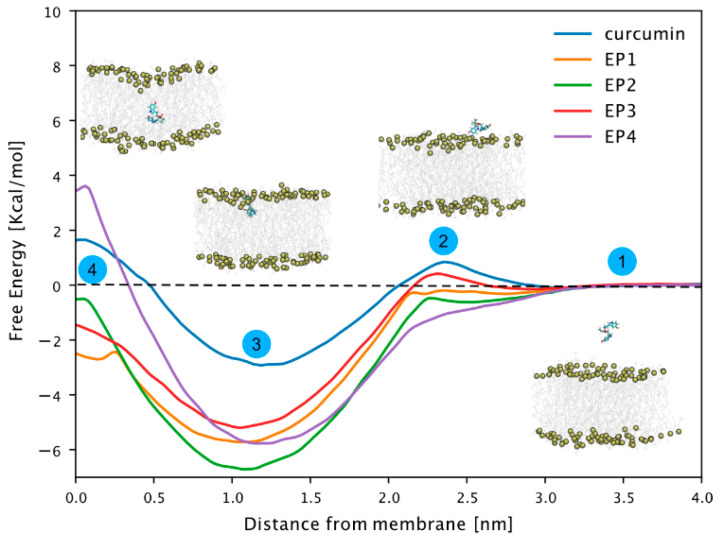
Free energy profiles of **EP1**–**EP4** and curcumin-related compounds. Potential of mean force (PMF) along with the z component of the distance between the center of masses of the compound and the P atoms of phospholipids in the closest leaflet. A total of 50 windows with 0.1 nm spacing were simulated for 100 ns. The first 50 ns were discarded from the PMF calculation and considered as equilibration time. The reconstructed free energy profiles’ statistical errors were estimated with the Bayesian bootstrap method using 200 bootstrap iterations. The error bar is not visible as it is smaller than the thickness of the line itself.

We conducted free energy calculations using the umbrella sampling technique [33,34] to investigate the EPi compound’s membrane permeabilization compared to curcumin. This allowed us to determine the free energy profile for transferring these compounds from the aqueous phase to the center of a model 1-palmitoyl-2-oleoly-*sn*-glycero-3-phosphocholine (POPC) membrane, as illustrated in Figure 2, steps 1–4. All compounds exhibited a free energy minimum at approximately 1 nm distance from the center of the membrane. At this position (step 3, Figure 2), the compounds’ center of mass resides slightly below the phosphorus atoms of the membrane lipids.

Notably, the free energy of all compounds decreased significantly in this region compared to that of curcumin, likely due to a phosphate group like those found in POPC lipid molecules. There was no significant barrier to transfer from the water phase to the phosphorus layer of the membrane. Only curcumin and **EP3** showed a slight barrier, but it was within the range of thermal fluctuations. Notably, while the free energy generally shifted towards more positive values near the bilayer’s center of mass (step 4, Figure 2), only curcumin and **EP4** exhibited positive free energy. This indicates unfavorable conditions within the membrane’s hydrophobic core. In conclusion, our study suggests that while membrane permeabilization is not a favored process, compounds tend to associate with the membrane, particularly at the water–lipid interface region.

### 3.4. Cytotoxic Effect on Different Cancer Cell Lines

**Figure 3:**
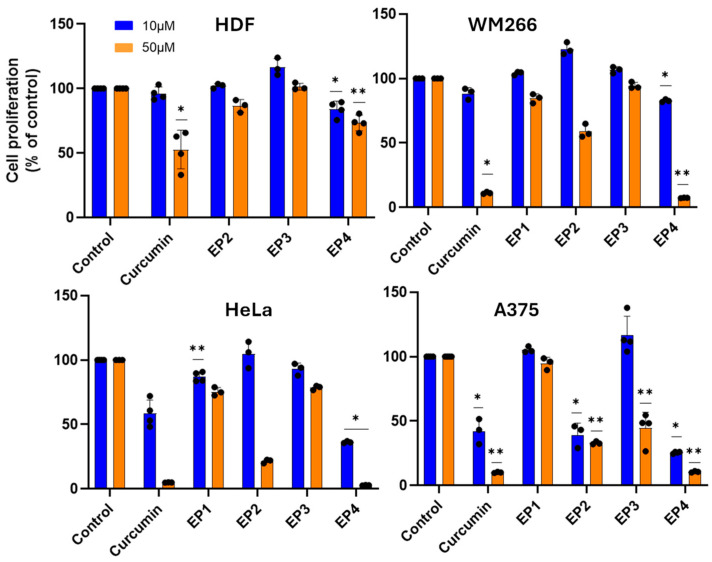
Cytotoxic activity of compounds against malignant and healthy cells. Cells were incubated in the presence of the compounds at 10 µM or 50 µM for 48 h, and the cytotoxic activity was determined by MTT assay. The results are presented as the percentage of proliferating cells relative to the control (0.5% DMSO-treated cells) and are expressed as means ± SE, * *p* < 0.05. ** *p* < 0.01.

**Figure 4:**
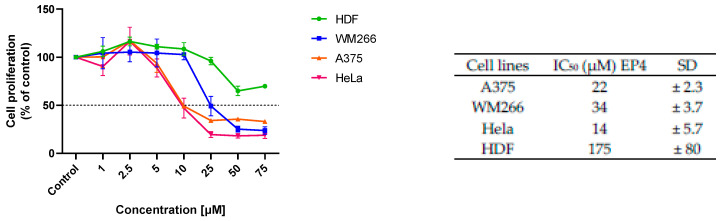
Concentration dependency of the cytotoxic effect of EP4 on different cell lines. The results are presented as the percentage of proliferating cells compared to the control (vehicle-treated cells) and are expressed as means ± SD of three independent experiments performed in duplicate, IC_50_ (µM) values (±SD) obtained after 48 h of incubation.

The cytotoxic activity of EPs was firstly screened on human A375 and WM266 melanoma cells and human HeLa cervix carcinoma cells (Figure 3). As normal cells, human dermal fibroblasts (HDFs) were chosen to estimate the selectivity of the tested compounds towards cancer cells. When cells were treated with 10 and 50 µM of the compounds for 48 h, all showed relevant cytotoxic activity on the cancer cell lines studied, except for **EP3**, which was inactive. Concentration-dependent studies showed significant cytotoxicity of **EP4** in all cancer cell lines, with EC_50_ values in the range of 15–35 µM, and marked selectivity for cancer cells, with an EC_50_ of 175 µM on healthy HDF cells (Figure 4).

**Figure 5:**
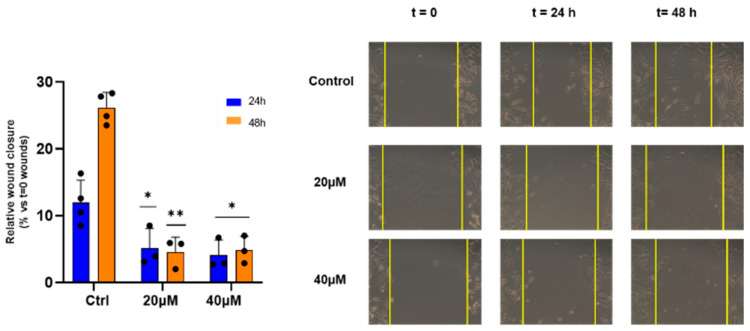
Effect of **EP4** on wound healing activity. WM266 cells were scratched and treated with **EP4** at the indicated concentrations. Left panel: bars depict mean ± SE of three independent experiments. Right panel: representative images of wound healing at 0 h, 24 h, and 48 h are shown. * *p* values < 0.05, calculated towards the control, were statistically significant; ** *p* < 0.01.

Furthermore, the ability of **EP4** to inhibit metastatic cell migration was studied. Therefore, WM266 monolayers were linearly scratched and incubated with **EP4** at both concentrations, one corresponding to about EC_50_ and the other corresponding to half of its value to avoid the high mortality of the cells assayed at 48 h, the end point of this assay. **EP4** (20 and 40 µM) significantly reduced the migration of WM266 cells, as measured by decreased wound closure within 24 to 48 h (Figure 5).

**Figure 6:**
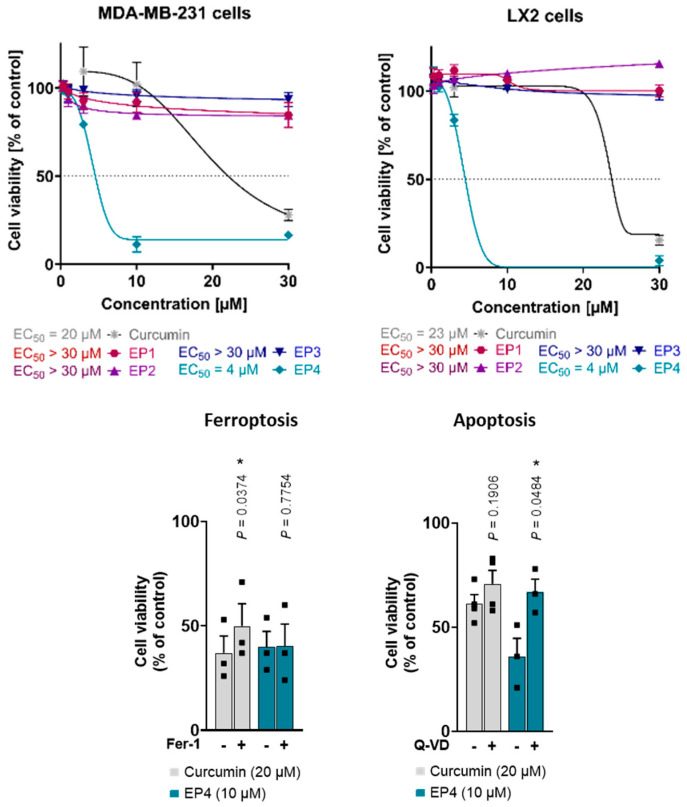
(***Top***): Effects of curcumin and EPs on the viability of human MDA-MB-231 breast cancer and human LX2 stellate cells. Cells were treated with vehicle (0.5% DMSO) or compounds at different concentrations (0.3, 1, 3, 10, and 30 μM) for 48 h. Cell viability measured by MTT assay. Asymmetric sigmoidal (5 PL) curve fit was used. Individual values and mean ± SEM of *n* = 3 independent experiments. (***Bottom***): Curcumin induces both ferroptosis and apoptosis, whereas **EP4** only induces apoptosis. Human MDA-MB-231 breast cancer cells were treated with vehicle (1% DMSO), curcumin, or **EP4** in combination with the ferroptosis inhibitor ferrostatin-1 (Fer-1, 10 μM) or the apoptosis inhibitor Q-VD-Oph (Q-VD, 50 μM) and incubated for 48 h. Cell viability was determined by MTT assay. Individual values and mean + SEM of *n* = 3 (Fer-1, **EP4** + Q-VD) and *n* = 4 (curcumin + Q-VD) independent experiments. * *p* < 0.05; two-sided paired Student’s *t*-test.

Moreover, curcumin and the EPs were evaluated for their cytotoxic properties in human triple-negative MDA-MB-231 breast cancer cells with a mesenchymal-like phenotype and human LX2 stellate cells. Interestingly, only curcumin and **EP4** suppressed cellular metabolic activity (measured by MTT assay) at concentrations up to 30 µM (Figure 6, *top*), with the lethal activity of **EP4** (EC_50_ value of 4 µM in both cell lines) exceeding that of curcumin (MDA-MB-231: EC_50_ = 20 µM; LX2: EC_50_ = 23 µM). The other mimics did not show any cytotoxic effects. These findings are in line with what was observed for other cell lines, such as human A375 melanoma cells: **EP4** is the most active compound in reducing metabolic activity, whereas effects for the other mimetics were only evident at substantially higher concentrations.

The induction of apoptosis and ferroptosis by curcumin has been previously reported [5,27,35]. To determine the specific cell death pathway responsible for the observed cytotoxicity of **EP4**, curcumin and **EP4** were tested in combination with inhibitors of ferroptosis (Ferrostatin-1, Fer-1) and apoptosis (Quinoline-Val-Asp-difluorophenoxymethylketone, Q-VD). Consistent with the literature, the results showed that curcumin-induced cell death was partially attenuated by Fer-1 and tendentially by Q-VD, suggesting a mixed type of cell death (Figure 6, *bottom*) [5]. Meanwhile, the cytotoxic effects of **EP4** were mitigated by Q-VD and remained unaffected by Fer-1 (Figure 6, *bottom*). This suggests that **EP4**-induced cell death involves apoptosis but not ferroptosis.

### 3.5. Effects of Ethylphosphonates **EP1**–**EP4** on Ubiquitin Activation

Ubiquitylation serves vital functions in protein quality control, with significant implications for signal transduction, DNA repair, cell growth, and apoptosis. This essential cellular mechanism comprises a series of enzymatic actions involving three key enzymes: the ubiquitin-activating enzyme (E1), which initiates the process by activating ubiquitin; the ubiquitin-conjugating enzyme (E2), which transfers ubiquitin from E1 to the target protein; and the ubiquitin protein ligase (E3), which imparts specificity by recognizing and binding to particular substrates [26]. Recent investigations have supported the role of E1 enzymes in the progression of carcinogenesis [36].

Over 40 inhibitors have been developed to target specific E1 enzymes, including those involved in ubiquitylation (UBA1), SUMOylation (SAE), and neddylation (NAE) [37]. Understanding E1 biology and developing selective inhibitors provide promising avenues for advancing cancer treatment. As an example, PYR-41 is a cell-permeable, irreversible inhibitor of the ubiquitin-activating enzyme E1. It prevents the activation of E1, disrupting the initial step of ubiquitylation.

Several cellular effects were observed after the treatment with PYR-41, including the inhibition of NF-κB activation, increased levels and activity of p53, and induction of apoptosis. Recently, the effect of curcumin on the thiol-ester bond formation between UBE1L (E1-like protein) and ISG15 (ubiquitin-like protein) has been explored [35], demonstrating that curcumin was able to prevent its formation. However, the potential influence on the formation of the thioester linkage between Ub and E1 has not been investigated yet. Thus, we planned to verify whether E1 enzyme activity could be influenced by ethylphosphonates (**EP1**–**EP4**), using a spectrophotometric assay for pyrophosphate determination. Pyrophosphate release was directly correlated with the formation of the thioester bond Ub-E1, as described in the experimental section. Screening of **EP1**–**EP4** compounds at 5 µM showed that only **EP4** inhibits E1 enzyme activity (Figure S3 in Supporting Information).

**Figure 7:**
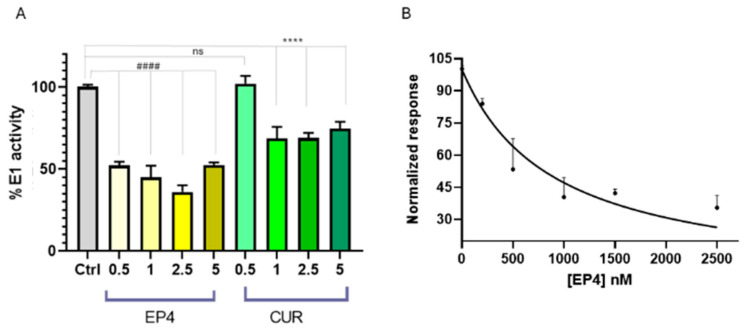
(**A**) E1 enzyme activity in the presence of **EP4** (yellow bars) and curcumin (green bars) at concentrations of 0.5–5 μM, using Ub (80 μM), E1 (100 nM), and E2 (300 nM) in tris buffer. E1 activity is expressed as a percentage relative to a control (gray bar) without ligands. Statistical significance is indicated as #### or **** (*p* < 0.0001 for **EP4** and curcumin, respectively). (**B**) A least-squares regression analysis of [**EP4**] versus normalized E1 inhibition percentage yielded R^2^ = 0.95. Data points and error bars represent the mean ± SD from two independent experiments in quadruplicate.

To compare the results for **EP4** with those for curcumin, different concentrations of **EP4** or curcumin were incubated in the presence of ubiquitin, the E1 enzyme, and activating buffer to monitor the release of pyrophosphate ions, as previously described [38]. **EP4** was able to inhibit the E1 enzyme during ubiquitin activation, with greater potency compared to curcumin. (Figure 7A). The half-maximal inhibitory concentration (IC_50_) was determined using a nonlinear fit, as shown in Figure 7B. The calculated value was 0.89 ± 0.07 µM, confirming that **EP4** is a promising inhibitor of E1 and encouraging further investigations into its potential therapeutic use in cancer treatment.

### 3.6. Protection Against RSL3-Induced Ferroptotic Cell Death

**Figure 8:**
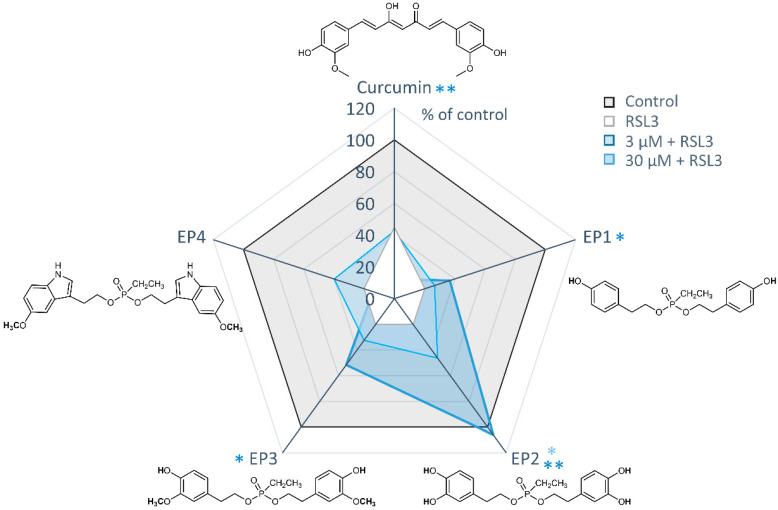
Protective effects of EP curcumin mimics against RSL3-induced ferroptosis. Radar plot illustrating cell viability measured by MTT assay. Human MDA-MB-231 breast cancer cells were treated with vehicle (1% DMSO), RSL3 (0.3 µM), or curcumin or ethylphosphonates (3 or 30 μM) in combination with RSL3 (0.3 µM). Data are presented as the mean of n = 3 independent experiments. * *p* < 0.05, ** *p* < 0.01; paired Student’s *t*-test.

To assess the potential cytoprotective properties of EP derivatives against ferroptosis, all compounds were tested at concentrations of 3 and 30 μM, either alone or in combination with the GPX4 inhibitor RSL3 (Figure 8).

Among the mimics tested, **EP2** showed the most potent protective effects against RSL3-induced ferroptosis. It significantly inhibited ferroptotic cell death at a concentration of 3 μM and completely prevented ferroptosis at 30 μM. Weak but significant protective effects were also evident for the derivatives **EP1** and **EP3** at 30 μM, leading to an increase in cell viability of about 20%. Conversely, the two cytotoxic compounds, curcumin and **EP4**, did not exhibit cytoprotective effects at either 3 or 30 μM.

### 3.7. Investigating the Ability of Ethylphosphonates (EPis) to Inhibit the Aggregation of Aβ Peptides

Alzheimer’s disease (AD) is the predominant form of age-related dementia, characterized by a progressive decline in memory and cognitive abilities [39]. A key pathological hallmark of AD is the presence of extracellular aggregates of amyloid β (Aβ) peptides in senile plaques [40]. Dysregulated processing of the amyloid precursor protein (APP) by β- and λ-secretases in the AD brain can lead to elevated Aβ peptide levels, eventually leading to abnormal accumulation in neurons and cell death [41]. Consequently, inhibition of Aβ self-assembly appears to be a promising therapeutic option for AD, with various compounds, including those of natural origin, being investigated for their anti-aggregation properties [42,43,44,45,46].

**Figure 9:**
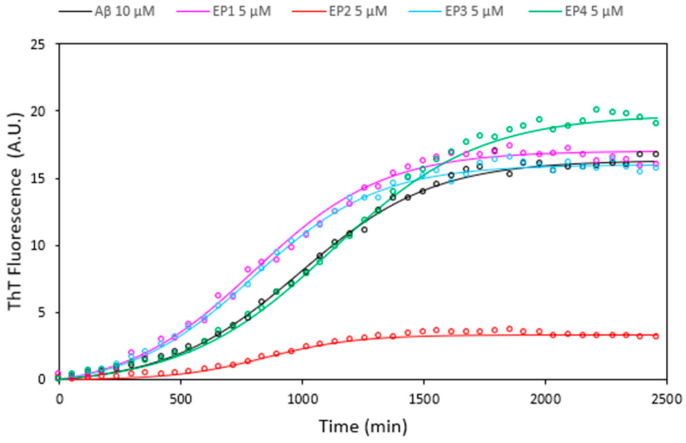
Thioflavin T fluorescence assays illustrating the kinetics of Aβ 10 µM fibril formation alone (black curve) and in the presence of **EP1** (pink curve), **EP2** (red curve), **EP3** (cyan curve), and **EP4** (green curve) at a concentration of 5 µM. Data acquired at 37 °C in 10 mM phosphate buffer, 100 mM NaCl, pH = 7.4, represent the average of three experiments.

Curcumin and its analogues have received great attention for their possible use in AD therapy in inhibiting amyloid peptide aggregation, which is a critical factor in the development of Alzheimer’s disease [47,48,49,50,51]. The effects of all EPs on Aβ amyloid growth were investigated by Thioflavin T (ThT) fluorescence assays at a 1:0.5 ligand: peptide molar ratio (Figure 9). The results unequivocally indicate that solely **EP2** exhibits notable inhibition of Aβ amyloid aggregation, while the remaining compounds displayed minimal activity.

## 4. Conclusions

In this study, novel curcumin mimics were designed and synthesized, aiming at improving the stability of the parent compound. The use of curcumin is limited due to its low water solubility in neutral conditions, high decomposition rate in alkaline media, and photodegradation in organic solvents. The 1, 3-dicarbonyl moiety of the original lead compound, curcumin, was replaced by a polar and non-charged ethyl phosphonate group. The length of the linker and the two aromatic moieties responsible for the curcumin activity were conserved. Curcumin is known to have cytotoxic effects and suppress cancer growth; these effects have been attributed to the induction of both apoptosis and ferroptosis. On the other hand, curcumin is also known for its cytoprotective properties and its ability to inhibit ferroptotic cell death [5].

This study outlines the synthesis and preliminary biological evaluation of new curcumin mimics, ethylphosphonates (**EP1**–**EP4**). The synthesis of EPs was conducted with high efficiency through orthogonal protection of building block groups. As a result, the tested compounds showed sufficient water solubility, good stability, and superior drug-like properties compared to curcumin, suggesting a more effective performance in vivo. The compound **EP2** showed high antioxidant activity in both ORAC and DPPH assays.

Among curcumin mimics, we have identified compounds that outperform curcumin in either cytotoxic (**EP4**) or cytoprotective (**EP2**) properties. **EP4** exhibited significant cytotoxicity in various human cancer cell lines (HeLa, A375, WM266) while sparing normal cells (HDFs), displaying a significant ability to reduce the migration of metastatic WM266 cells. Additionally, **EP4** displayed five-times-higher toxicity in MDA-MB-231 and LX2 cells than curcumin, demonstrating a mechanism of cell mortality that is more dependent on apoptosis compared to curcumin and involves both ferroptotic and apoptotic components. Accordingly, **EP4** is a more potent inhibitor of the ubiquitin-activating enzyme E1 than curcumin. In contrast**, EP2** exhibits a significant ability to protect cells against the ferroptosis mechanism, completely preventing ferroptosis at 30 μM. Interestingly, **EP2** was also found to be more effective in the inhibition of amyloid peptide aggregation, a hallmark of Alzheimer’s disease. Altogether, **EP4** stands out in combating various types of cancer, whereas **EP2** shows promise for treating neurodegenerative disorders. Although the number of mimics is limited, the reported results are a good starting point for further expansions of the EP mimics library to allow a clear structure–activity relationship.

## Figures and Tables

**Table 1:** Yields, solubility, and antioxidant activity (ORAC and DPPH) for **EP1**–**EP4**.

Compound	Yield (%)	ORAC (TE)	DPPH (EC_50_, µM)	Solubility (μM) ^a^
Curcumin	–	5.03 ± 0.14	13.5 ± 0.50	Insoluble [31]
TYR	–	2.18 ± 0.12	>1000	–
MTYR	–	2.90 ± 0.21	31.0 ± 2.6	–
HTYR	–	7.40 ± 0.17	12.3 ± 1.0	–
MEL	–	2.23 ± 0.12	>1000	–
**EP1**	60	5.12 ± 0.56	>1000	15.0 ± 0.23
**EP2**	75	8.84 ± 0.45	6.00 ± 0.43	40.0 ± 0.58
**EP3**	48	4.14 ± 0.29	17.22 ± 0.08	40.0 ± 0.49
**EP4**	34	7.24 ± 0.46	>1000	20.0 ± 0.21

TE: Trolox equivalent; ^a^ experimental details, see Supplementary Materials.

## Data Availability

Data supporting the results are reported in the Supplementary Materials.

## Supplementary Materials

The following supporting information can be downloaded at https://www.mdpi.com/article/10.3390/antiox14040412/s1. Material and Instruments, Medium stability, Water solubility measurements, DPPH assay, ORAC assay and NMR spectra.

## References

[1] Newman D.J., Cragg G.M. (2020). Natural Products as Sources of New Drugs over the Nearly Four Decades from 01/1981 to 09/2019. J. Nat. Prod..

[2] Choudhary S., Singh P.K., Verma H., Singh H., Silakari O. (2018). Success Stories of Natural Product-Based Hybrid Molecules for Multi-Factorial Diseases. Eur. J. Med. Chem..

[3] Zhou Z., Li J., Zhang X. (2023). Natural Flavonoids and Ferroptosis: Potential Therapeutic Opportunities for Human Diseases. J. Agric. Food Chem..

[4] Gali-Muhtasib H., Hmadi R., Kareh M., Tohme R., Darwiche N. (2015). Cell Death Mechanisms of Plant-Derived Anticancer Drugs: Beyond Apoptosis. Apoptosis.

[5] Koeberle S.C., Kipp A.P., Stuppner H., Koeberle A. (2023). Ferroptosis-Modulating Small Molecules for Targeting Drug-Resistant Cancer: Challenges and Opportunities in Manipulating Redox Signaling. Med. Res. Rev..

[6] Devisscher L., Van Coillie S., Hofmans S., Van Rompaey D., Goossens K., Meul E., Maes L., De Winter H., Van Der Veken P., Vandenabeele P. (2018). Discovery of Novel, Drug-Like Ferroptosis Inhibitors with in Vivo Efficacy. J. Med. Chem..

[7] Dixon S.J., Lemberg K.M., Lamprecht M.R., Skouta R., Zaitsev E.M., Gleason C.E., Patel D.N., Bauer A.J., Cantley A.M., Yang W.S. (2012). Ferroptosis: An Iron-Dependent Form of Nonapoptotic Cell Death. Cell.

[8] Ryan S.K., Ugalde C.L., Rolland A.S., Skidmore J., Devos D., Hammond T.R. (2023). Therapeutic Inhibition of Ferroptosis in Neurodegenerative Disease. Trends Pharmacol. Sci..

[9] Wang Y., Wu S., Li Q., Sun H., Wang H. (2023). Pharmacological Inhibition of Ferroptosis as a Therapeutic Target for Neurodegenerative Diseases and Strokes. Adv. Sci..

[10] Zhang S., Hu R., Geng Y., Chen K., Wang L., Imam M.U. (2021). The Regulatory Effects and the Signaling Pathways of Natural Bioactive Compounds on Ferroptosis. Foods.

[11] Gu F., Zhu M., Shi J., Hu Y., Zhao Z. (2008). Enhanced Oxidative Stress Is an Early Event during Development of Alzheimer-like Pathologies in Presenilin Conditional Knock-out Mice. Neurosci. Lett..

[12] Lesjak M., Simin N., Srai S.K.S. (2022). Can Polyphenols Inhibit Ferroptosis?. Antioxidants.

[13] Kajarabille N., Latunde-Dada G.O. (2019). Programmed Cell-Death by Ferroptosis: Antioxidants as Mitigators. Int. J. Mol. Sci..

[14] Esatbeyoglu T., Huebbe P., Ernst I.M.A., Chin D., Wagner A.E., Rimbach G. (2012). Curcumin-From Molecule to Biological Function. Angew. Chem. Int. Ed..

[15] Patel S.S., Acharya A., Ray R.S., Agrawal R., Raghuwanshi R., Jain P. (2020). Cellular and Molecular Mechanisms of Curcumin in Prevention and Treatment of Disease. Crit. Rev. Food Sci. Nutr..

[16] Mortezaee K., Salehi E., Mirtavoos-mahyari H., Motevaseli E., Najafi M., Farhood B., Rosengren R.J., Sahebkar A. (2019). Mechanisms of Apoptosis Modulation by Curcumin: Implications for Cancer Therapy. J. Cell. Physiol..

[17] El-Saadony M.T., Yang T., Korma S.A., Sitohy M., El-Mageed T.A.A., Selim S., Al Jaouni S.K., Salem H.M., Mahmmod Y., Soliman S.M. (2023). Impacts of Turmeric and Its Principal Bioactive Curcumin on Human Health: Pharmaceutical, Medicinal, and Food Applications: A Comprehensive Review. Front. Nutr..

[18] Nie A., Shen C., Zhou Z., Wang J., Sun B., Zhu C. (2024). Ferroptosis: Potential Opportunities for Natural Products in Cancer Therapy. Phytother. Res..

[19] Thapa A., Jett S.D., Chi E.Y. (2016). Curcumin Attenuates Amyloid-β Aggregate Toxicity and Modulates Amyloid-β Aggregation Pathway. ACS Chem. Neurosci..

[20] Nelson K.M., Dahlin J.L., Bisson J., Graham J., Pauli G.F., Walters M.A. (2017). The Essential Medicinal Chemistry of Curcumin. J. Med. Chem..

[21] Bahadori F., Demiray M. (2017). A Realistic View on “the Essential Medicinal Chemistry of Curcumin”. ACS Med. Chem. Lett..

[22] Zhou H., Beevers C.S., Huang S. (2012). The Targets of Curcumin. Curr. Drug Targets.

[23] Zhao S., Pi C., Ye Y., Zhao L., Wei Y. (2019). Recent Advances of Analogues of Curcumin for Treatment of Cancer. Eur. J. Med. Chem..

[24] Costantino M., Corno C., Colombo D., Perego P. (2022). Curcumin and Related Compounds in Cancer Cells: New Avenues for Old Molecules. Front. Pharmacol..

[25] Reinke A.A., Gestwicki J.E. (2007). Structure-Activity Relationships of Amyloid Beta-Aggregation Inhibitors Based on Curcumin: Influence of Linker Length and Flexibility. Chem. Biol. Drug Des..

[26] Romanucci V., Giordano M., De Tommaso G., Iuliano M., Bernini R., Clemente M., Garcia-Viñuales S., Milardi D., Zarrelli A., Di Fabio G. (2021). Synthesis of New Tyrosol-Based Phosphodiester Derivatives: Effect on Amyloid β Aggregation and Metal Chelation Ability. ChemMedChem.

[27] Romanucci V., Giordano M., Pagano R., Agarwal C., Agarwal R., Zarrelli A., Di Fabio G. (2021). Solid-Phase Synthesis of Curcumin Mimics and Their Anticancer Activity against Human Pancreatic, Prostate, and Colorectal Cancer Cell Lines. Bioorganic Med. Chem..

[28] Su F., Descher H., Bui-Hoang M., Stuppner H., Skvortsova I., Rad E.B., Ascher C., Weiss A., Rao Z., Hohloch S. (2024). Iron(III)-Salophene Catalyzes Redox Cycles That Induce Phospholipid Peroxidation and Deplete Cancer Cells of Ferroptosis-Protecting Cofactors. Redox Biol..

[29] Gollowitzer A., Pein H., Rao Z., Waltl L., Bereuter L., Loeser K., Meyer T., Jafari V., Witt F., Winkler R. (2025). Attenuated Growth Factor Signaling during Cell Death Initiation Sensitizes Membranes towards Peroxidation. Nat. Commun..

[30] Di Gaetano S., Pirone L., Galdadas I., Traboni S., Iadonisi A., Pedone E., Saviano M., Gervasio F.L., Capasso D. (2022). Design, Synthesis, and Anticancer Activity of a Selenium-Containing Galectin-3 and Galectin-9N Inhibitor. Int. J. Mol. Sci..

[31] Mangolim C.S., Moriwaki C., Nogueira A.C., Sato F., Baesso M.L., Neto A.M., Matioli G. (2014). Curcumin-β-Cyclodextrin Inclusion Complex: Stability, Solubility, Characterisation by FT-IR, FT-Raman, X-ray Diffraction and Photoacoustic Spectroscopy, and Food Application. Food Chem..

[32] Tønnesen H.H., Másson M., Loftsson T. (2002). Studies of Curcumin and Curcuminoids. XXVII. Cyclodextrin Complexation: Solubility, Chemical and Photochemical Stability. Int. J. Pharm..

[33] Torrie G.M., Valleau J.P. (1974). Monte Carlo Free Energy Estimates Using Non-Boltzmann Sampling: Application to the Sub-Critical Lennard-Jones Fluid. Chem. Phys. Lett..

[34] Torrie G.M., Valleau J.P. (1977). Nonphysical Sampling Distributions in Monte Carlo Free-Energy Estimation: Umbrella Sampling. J. Comput. Phys..

[35] Chan W.H., Wu H.Y., Chang W.H. (2006). Dosage Effects of Curcumin on Cell Death Types in a Human Osteoblast Cell Line. Food Chem. Toxicol..

[36] Yang Y., Kitagaki J., Dai R.M., Yien C.T., Lorick K.L., Ludwig R.L., Pierre S.A., Jensen J.P., Davydov I.V., Oberoi P. (2007). Inhibitors of Ubiquitin-Activating Enzyme (E1), a New Class of Potential Cancer Therapeutics. Cancer Res..

[37] Barghout S.H., Schimmer A.D. (2021). E1 Enzymes as Therapeutic Targets in Cancer. Pharmacol. Rev..

[38] Murai Y., Jo U., Murai J., Jenkins L.M., Huang S.-Y.N., Chakka S., Chen L., Cheng K., Fukuda S., Takebe N. (2021). SLFN11 Inactivation Induces Proteotoxic Stress and Sensitizes Cancer Cells to Ubiquitin Activating Enzyme Inhibitor TAK-243. Cancer Res..

[39] Selkoe D.J. (1991). The Molecular Pathology of Alzheimer’s Disease. Neuron.

[40] Coria F., Rubio I., Bayon C. (1994). Alzheimer’s Disease, ß-Amyloidosis, and Aging. Rev. Neurosci..

[41] Hamley I.W. (2012). The Amyloid Beta Peptide: A Chemist’s Perspective. Role in Alzheimer’s and Fibrillization. Chem. Rev..

[42] Ahmed R., Akcan M., Khondker A., Rheinstädter M.C., Bozelli J.C., Epand R.M., Huynh V., Wylie R.G., Boulton S., Huang J. (2019). Atomic Resolution Map of the Soluble Amyloid Beta Assembly Toxic Surfaces. Chem. Sci..

[43] Hyung S.J., Detoma A.S., Brender J.R., Lee S., Vivekanandan S., Kochi A., Choi J.S., Ramamoorthy A., Ruotolo B.T., Lim M.H. (2013). Insights into Antiamyloidogenic Properties of the Green Tea Extract (-)-Epigallocatechin-3-Gallate toward Metal-Associated Amyloid-β Species. Proc. Natl. Acad. Sci. USA.

[44] Lolicato F., Raudino A., Milardi D., La Rosa C. (2015). Resveratrol Interferes with the Aggregation of Membrane-Bound Human-IAPP: A Molecular Dynamics Study. Eur. J. Med. Chem..

[45] Romanucci V., García-Viñuales S., Tempra C., Bernini R., Zarrelli A., Lolicato F., Milardi D., Di Fabio G. (2020). Modulating Aβ Aggregation by Tyrosol-Based Ligands: The Crucial Role of the Catechol Moiety. Biophys. Chem..

[46] Sciacca M.F.M., Romanucci V., Zarrelli A., Monaco I., Lolicato F., Spinella N., Galati C., Grasso G., D’Urso L., Romeo M. (2017). Inhibition of Aβ Amyloid Growth and Toxicity by Silybins: The Crucial Role of Stereochemistry. ACS Chem. Neurosci..

[47] Hamaguchi T., Ono K., Yamada M. (2010). REVIEW: Curcumin and Alzheimer’s Disease. CNS Neurosci. Ther..

[48] Orlando R.A., Gonzales A.M., Royer R.E., Deck L.M., Jagt D.L.V. (2012). A Chemical Analog of Curcumin as an Improved Inhibitor of Amyloid Abeta Oligomerization. PLoS ONE.

[49] Rao P.P.N., Mohamed T., Teckwani K., Tin G. (2015). Curcumin Binding to Beta Amyloid: A Computational Study. Chem. Biol. Drug Des..

[50] Yanagisawa D., Taguchi H., Yamamoto A., Shirai N., Hirao K., Tooyama I. (2011). Curcuminoid Binds to Amyloid-Β1-42 Oligomer and Fibril. J. Alzheimer’s Dis..

[51] Kochi A., Lee H.J., Vithanarachchi S.M., Padmini V., Allen M.J., Lim M.H. (2015). Inhibitory Activity of Curcumin Derivatives Towards Metal-Free and Metal-Induced Amyloid-β Aggregation. Curr. Alzheimer Res..

