# Supplementary material for: Synthesis of Ethylphosphonate Curcumin Mimics: Substituents Allow Switching Between Cytotoxic and Cytoprotective Activities
Source: Antioxidants (Basel). 2025 Mar 29;14(4):412. doi: 10.3390/antiox14040412 (PMC12024457; doi:10.3390/antiox14040412)
Supplement: Supplementary file 1 [file antioxidants-14-00412-s001.zip › antioxidants-3544817-supplementary.pdf]

Supplementary Materials

Synthesis of ethylphosphonate curcumin mimics: substituents allow switching between cytotoxic and cytoprotective activities.

Valeria Romanucci[a], *Rita Pagano*[a], *Solveigh C. Koeberle*[b], *Andreas Koeberle*[b], *Minh Bui Hoang*[b], *Sonia Di Gaetano*[c], *Domenica Capasso*[d], *Michele Francesco Maria Sciacca*[e], *Valeria Lanza*[e], *Carmelo Tempra*[f], *Fabio Lolicato*[g,h], *Armando Zarrelli*[a], *Danilo Milardi*[e] and *Giovanni Di Fabio*[a]*

[a] Department of Chemical Sciences, University of Naples "Federico II", Complesso Monte Sant'Angelo, Via Cintia 4, I-80126 Napoli (NA), Italy

[b] Michael Popp Institute, Center for Molecular Biosciences Innsbruck (CMBI), University of Innsbruck, Tirol, Innsbruck, Austria

[c] Institute of Biostructures and Bioimaging, National Research Council (CNR), Via P. Castellino 111, Naples, 80131, Italy

[d] Department of Physics Ettore Pancini, University of Naples Federico II, Naples, Complesso Monte Sant'Angelo, Via Cintia 4, I-80126 Napoli (NA), Italy

[e] Istituto di Cristallografia, Consiglio Nazionale delle Ricerche, Via Paolo Gaifami 18, 95125 Catania, Italy

[f] Institute of Organic Chemistry and Biochemistry of the Czech Academy of Sciences, 160 00 Prague 6, Czech Republic

[g] Heidelberg University Biochemistry Center, Heidelberg, Germany

[h] Department of Physics, University of Helsinki, Helsinki, Finland

---

* corresponding author: Prof. Giovanni Di Fabio, Department of Chemical Sciences, University of Naples "Federico II", Complesso Monte Sant'Angelo, Via Cintia 4, I-80126 Napoli (NA), Italy; phone: 0039 081674001; *e*mail: difabio@unina.it;

Table of contents

pag.

Material and Instruments ……………………………………………………………… 3

Medium stability ……………………………………………………………………… 4

Water solubility measurements ……………………………………………………… 4

DPPH assay ……………………………………………………………………… 5

ORAC assay ……………………………………………………………………… 5

^{1}H, ^{31}P and ^{13}C NMR spectra for **EP1** ……………………………………………….. S3

^{1}H, ^{31}P and ^{13}C NMR spectra for **EP2** ……………………………………………….. S9

^{1}H, ^{31}P and ^{13}C NMR spectra for **EP3** ……………………………………………….. S15

^{1}H, ^{31}P and ^{13}C NMR spectra for **EP4** ……………………………………………….. S21

Figure SM1. Stability of **EP1** …………………………………………….. S27

Figure SM2. **EPs** water solubility …………………………………………….. S27

Figure SM3. Pyrophosphate release during E1 enzyme activation ……………………….. S28

Material and Instruments

All chemicals were purchased from Sigma–Aldrich (Milano, Italy). HPLC–grade ACN and MeOH were purchased from Carlo Erba Reagents and Sigma-Aldrich, respectively. Reactions were monitored by TLC (F254 precoated silica gel plates, Merck) and column chromatography (Merck Kieselgel 60, 70–230 mesh, Milano, Italy). HPLC analysis was performed with a Shimadzu LC–8A HPLC system (Shimadzu Analytical and Measuring Instruments, Milano, Italy) equipped with a Shimadzu SCL–10A VP System control and a Shimadzu SPD–10A VP UV-Vis detector. Mass spectrometric analyses were performed on AB SCIEX TOF/TOF 5800 in positive or negative mode and Waters Micromass ZQ Instrument (Waters, Milano, Italy) equipped with an electrospray source in positive mode. The NMR spectra were recorded at 25 °C on an NMR spectrometer Bruker DRX, Bruker Advance (Bruker Italia Srl, Milano, Italy) and INOVA-500 NMR instrument (Varian, Milan, Italy). Ubiquitin-activation enzyme (UBE1) and UbcH13/Uev heterodimer complex were obtained from Boston Biochem. Amyloid β peptide 1-40 (Aβ) of purity greater than 95% was provided by GenScript. Thioflavin T (ThT) was obtained from Sigma-Aldrich, while Hexafluoroisopropanol (HFIP) was sourced from Carlo Erba.

Medium stability

An appropriate amount of compound was dissolved in MilliQ water containing 0.5 % DMSO; the stability was carried out at 37 °C in PBS buffer (phosphate 10 mM, pH = 7.4, 100 mM NaCl) and in sGF fluid (sodium chloride was dissolved in MilliQ water (34.2 mM) then the pH was adjusted to 1.2 by adding 37% aq. HCl)[50]. Briefly, the compound was dissolved to a final concentration of 1 mM in the sGF and in PBS buffers. At different time points (ti, i = 0, 1, 3, 5, 24 hours), 100 µL was withdrawn from the reaction mixture, filtered, and injected into the HPLC system. HPLC analyses were carried out on a Phenomenex Luna RP18 column (5 µm particle size, 4.6 mm×150 mm i.d.) eluted in a gradient of B (CH_3CN) in A (0.1% HCOOH in H_2O) from 5% to 100% in 20 min at 0.8 mL/min (λ = 260 nm, 280 nm).

Water solubility measurements

For all the compounds were prepared stock solutions in DMSO in a concentration range of 5 mM-100 µM and then diluted in a cuvette with phosphate buffer 10 mM, pH = 7.4, to reach the final concentration range of 50-1 µM (1% DMSO). The UV spectra were recorded at λ = 200-600 nm for all ten concentrations. The absorbances at λ_{max} = 280 nm were plotted with the corresponding concentration, and the solubility limit was determined by considering the concentrations that fitted along the interpolation line with an R^2 of 0.9990.

DPPH assay

The DPPH solution (200 μM) was prepared in MeOH and allowed stand in the dark for 30 min before the analyses. Compounds were dissolved in MeOH to prepare stock solutions 100 μM. The DPPH solution was transferred to a cuvette (final concentration 50 μM), and the solutions of each compound (final concentration range 1 - 75 μM) were rapidly added and mixed into each test tube to achieve a final volume of 500 μL. The reaction was monitored by a continuous spectrophotometric analysis measuring the absorbance at λ = 517 nm for 30 min. The percentage of inhibition (% inhibition) was calculated using the equation:

$$\%\ inhibition = \frac{A_{control} - A_{sample}}{A_{control}}\ x\ 100$$

The EC_{50} value (the inhibition concentration of a sample at 50% fall in absorbance of DPPH) was used to compare the DPPH scavenging activities.

ORAC assay

150 μL of the fluorescein solution (11.12×10^{-2} μM in phosphate buffer 0.75 mM, pH = 7.4) was added into each well of a 96-well plate. Subsequently, 23 μL of buffer and 2 μL of stock solutions in DMSO of tested compounds were added to the wells to reach the final concentration range of 1.25-20 μM. The plate was incubated for 30 min at 37 °C, after which 25 μL of AAPH (152.6 mM) was added to each well. Immediately, the fluorescence was measured using a microplate reader for 2 hours with reading taken at 1 min intervals at 37 °C (λ_{ecc} = 485 nm, λ_{em} = 528 nm).

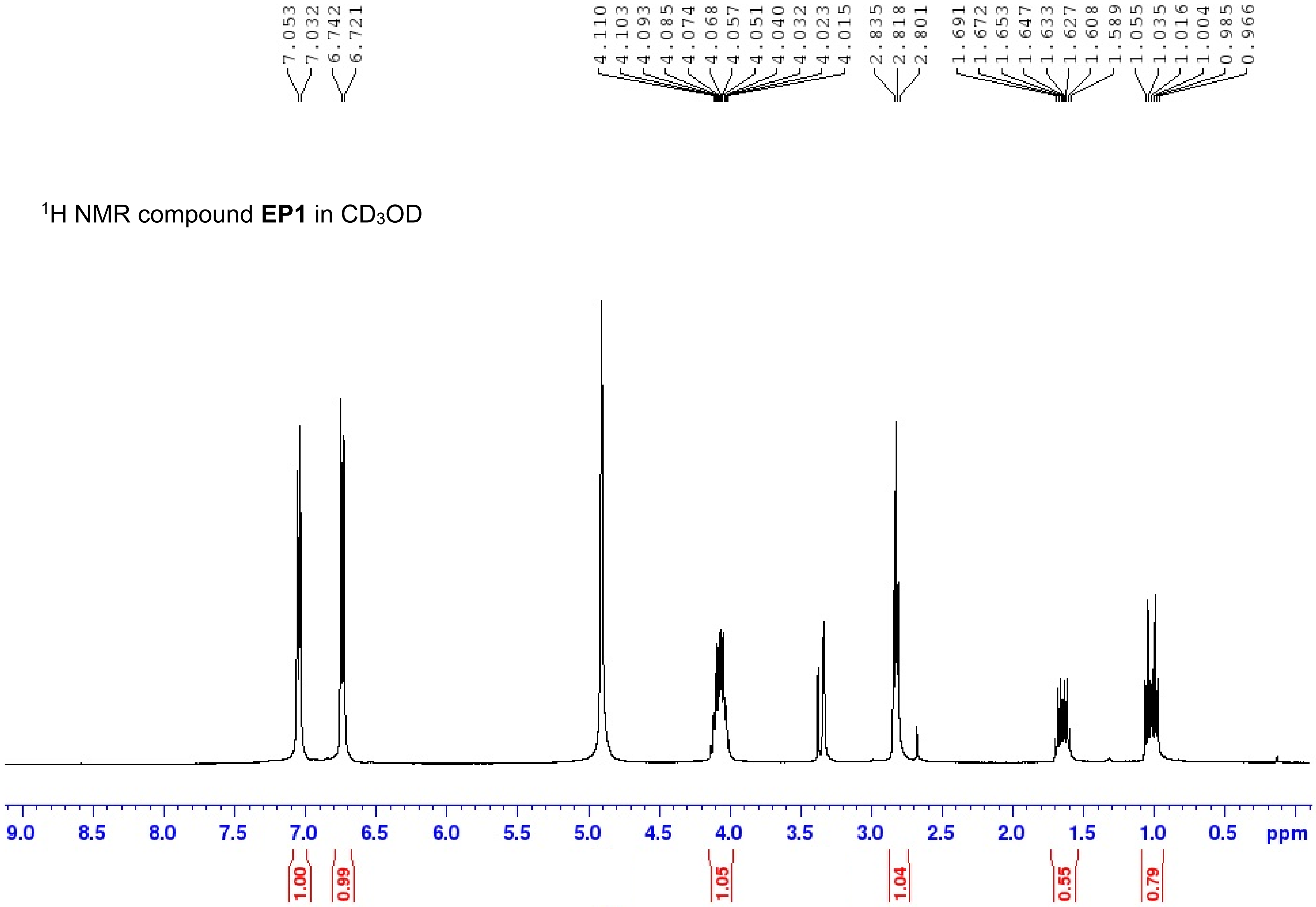

^{1}H NMR compound **EP1** in CD_3OD

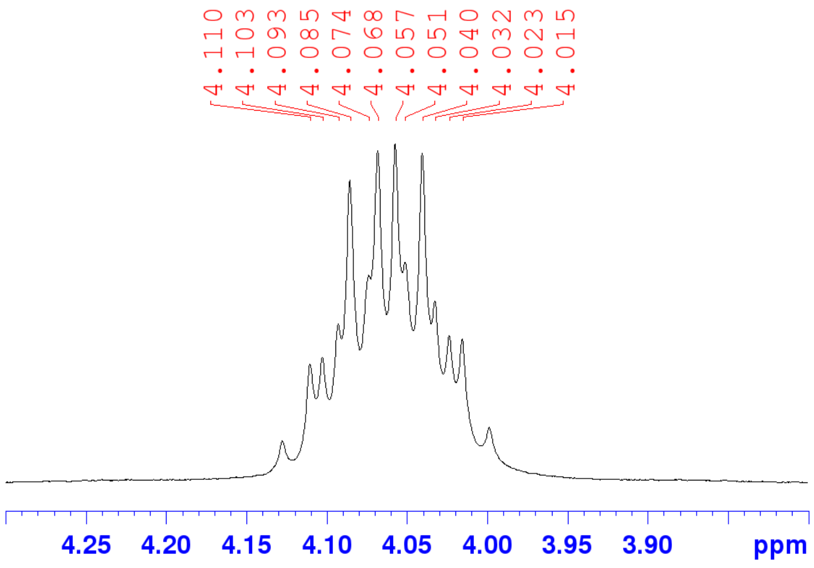

^{1}H NMR of **EP1** in CD_3OD: splitting pattern of H8 and H8' protons.

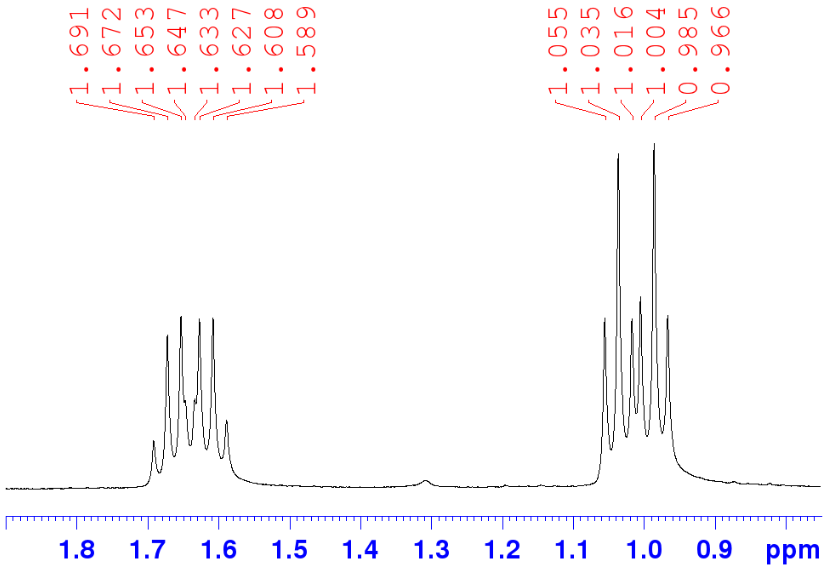

^{1}H NMR of **EP1** in CD_3OD: Splitting pattern of –CH_2CH_3 (AA'M_3X) protons.

^{31}P NMR proton decoupled of **EP1** in CD_3OD

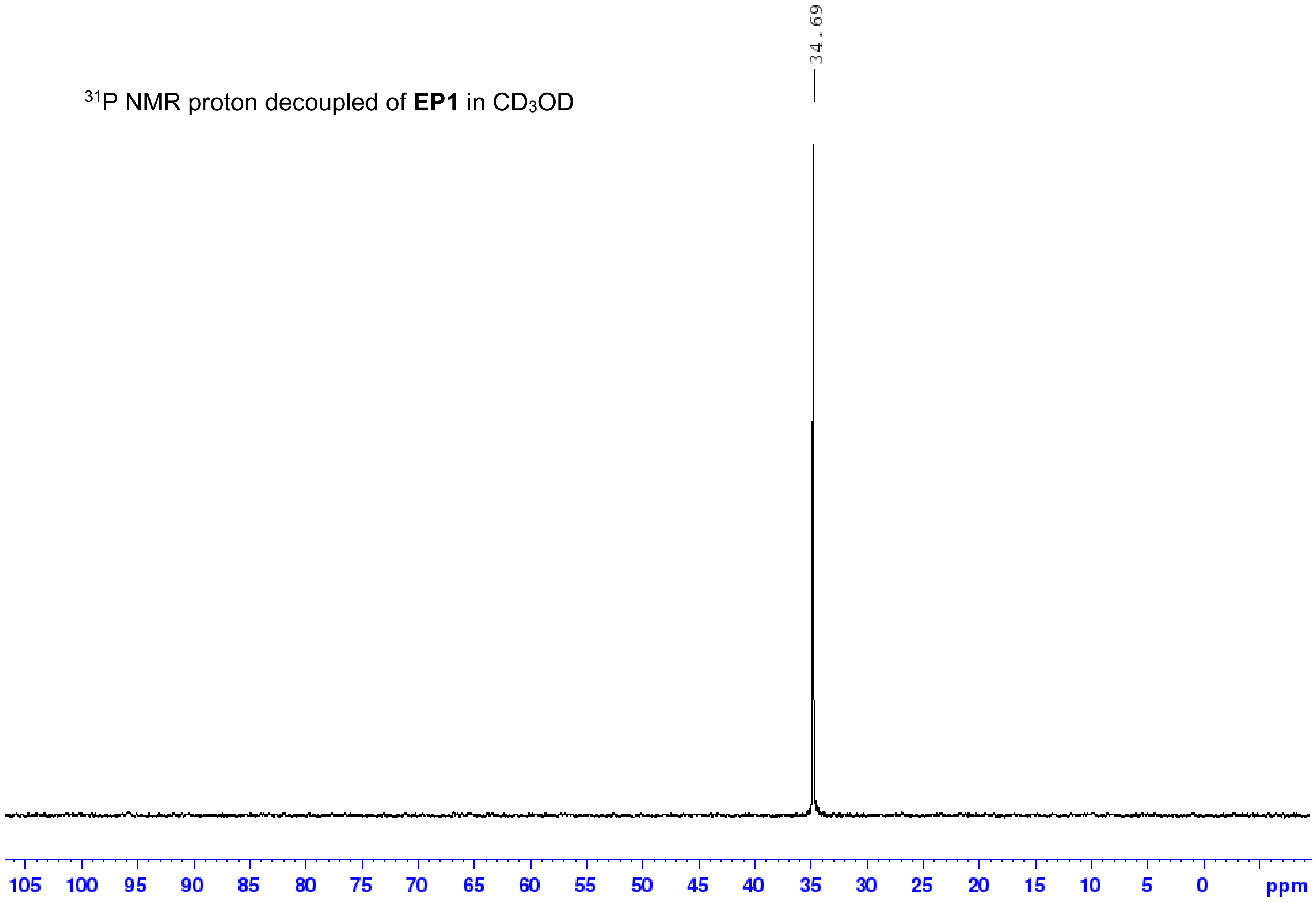

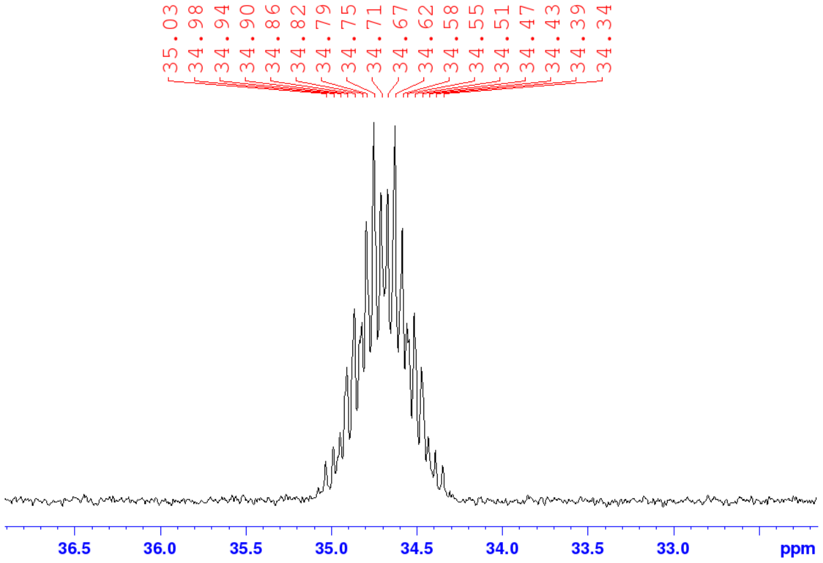

^{31}P NMR of **EP1** in CD_3OD

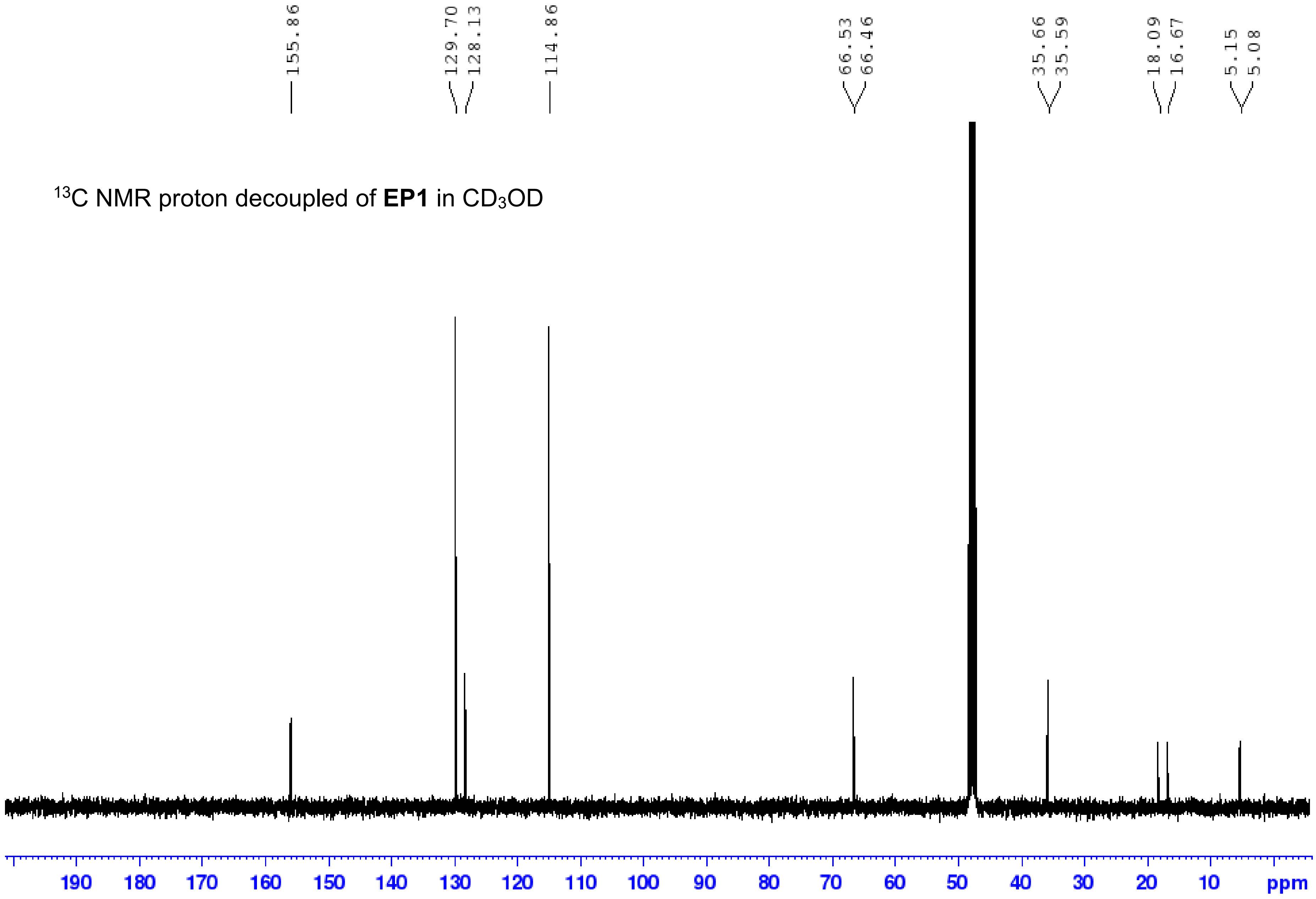

^{13}C NMR proton decoupled of **EP1** in CD_3OD

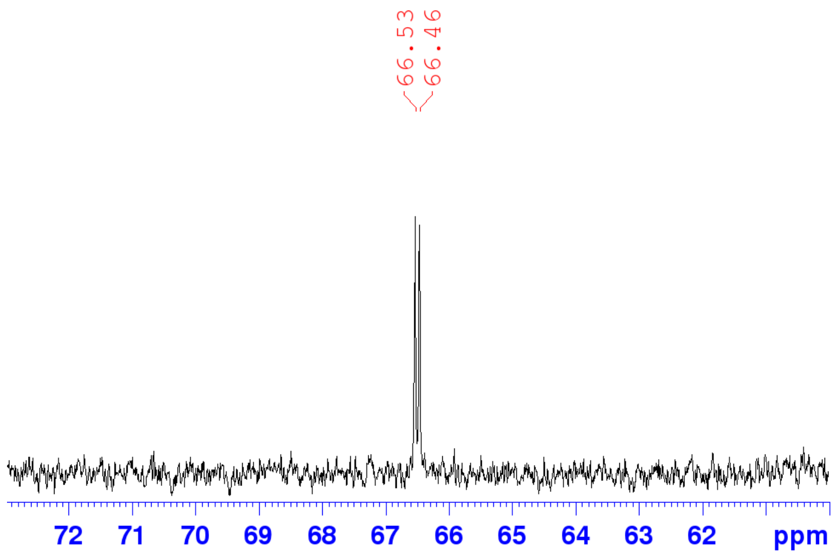

^{13}C NMR proton decoupled of **EP1** in CD_3OD: splitting pattern of C8 carbon.

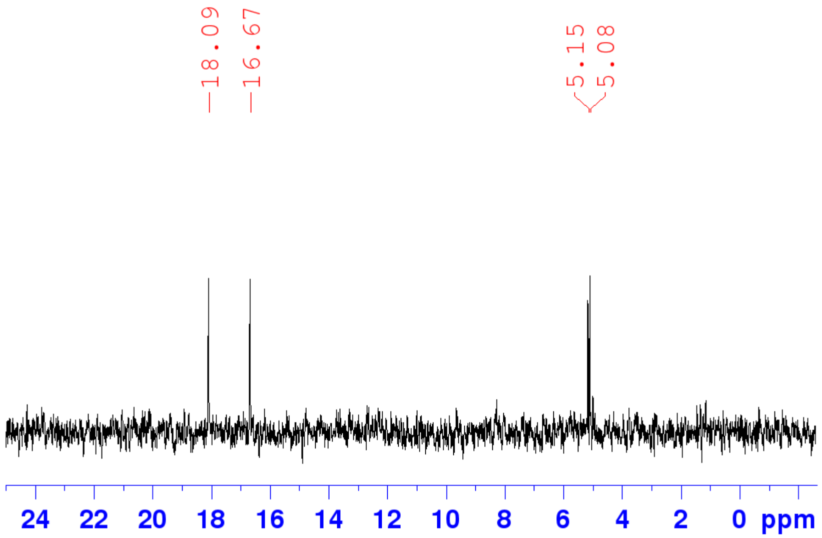

^{13}C NMR proton decoupled of **EP1** in CD_3OD: splitting pattern of $-CH_2CH_3$ carbons.

^{1}H NMR of **EP2** in CD_3OD

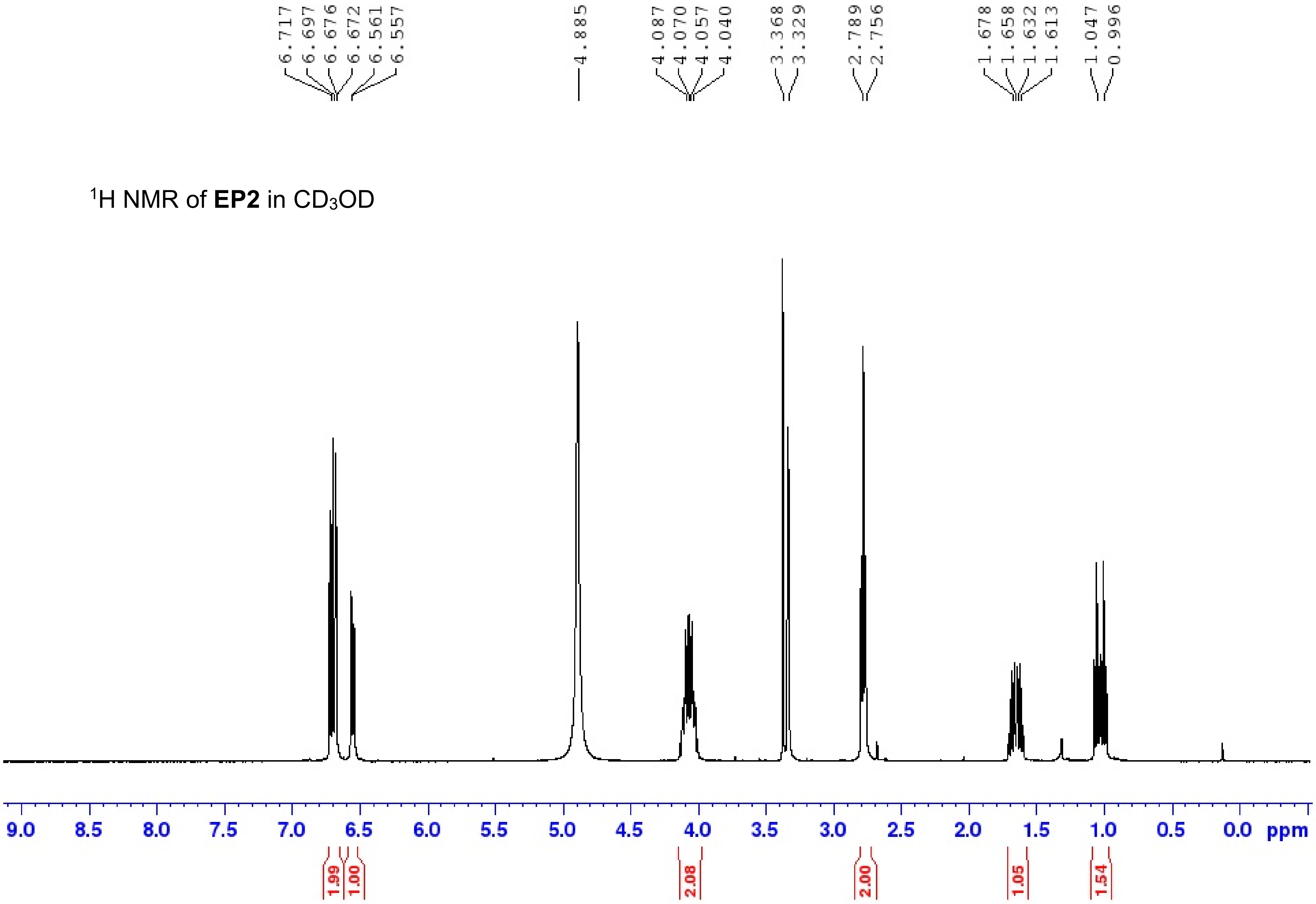

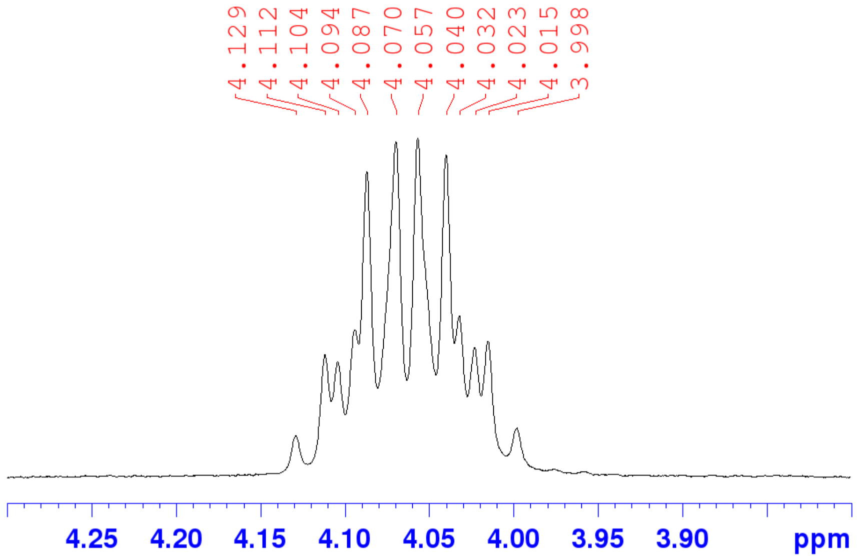

^{1}H NMR compound **EP2** in CD_3OD: splitting pattern of H8 and H8' protons.

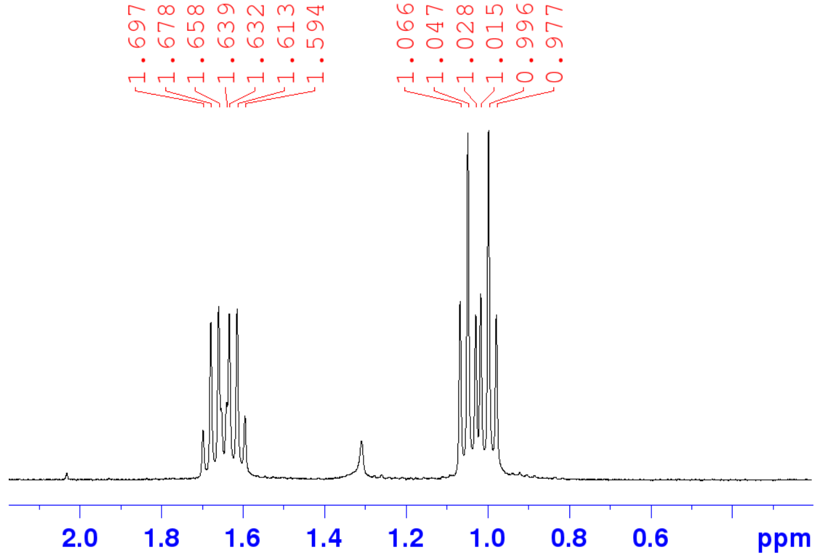

^{1}H NMR compound **EP2** in CD_3OD: Splitting pattern of $–CH_2CH_3$ (AA'M_3X) protons.

31P NMR proton decoupled of **EP2** in CD_3OD

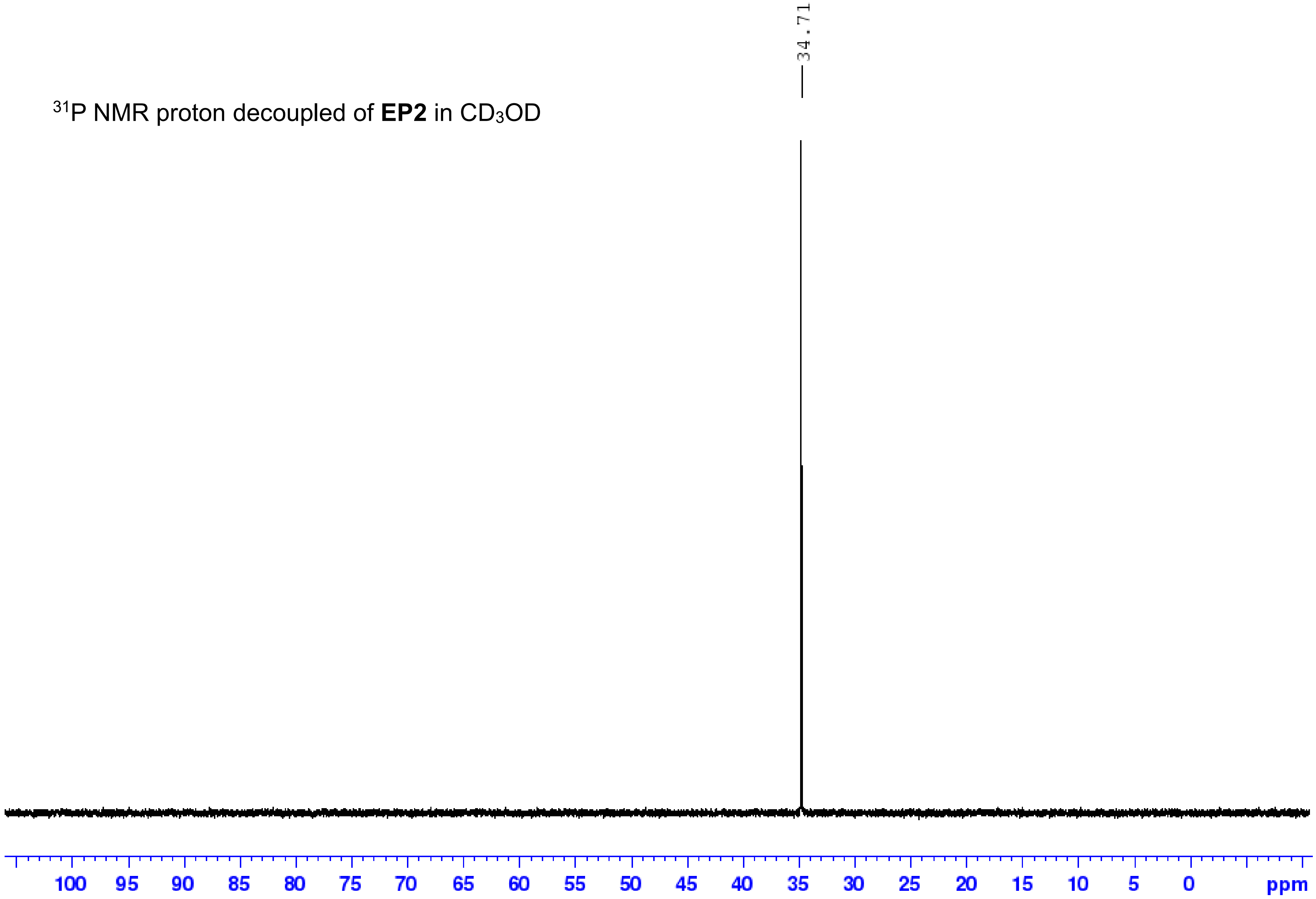

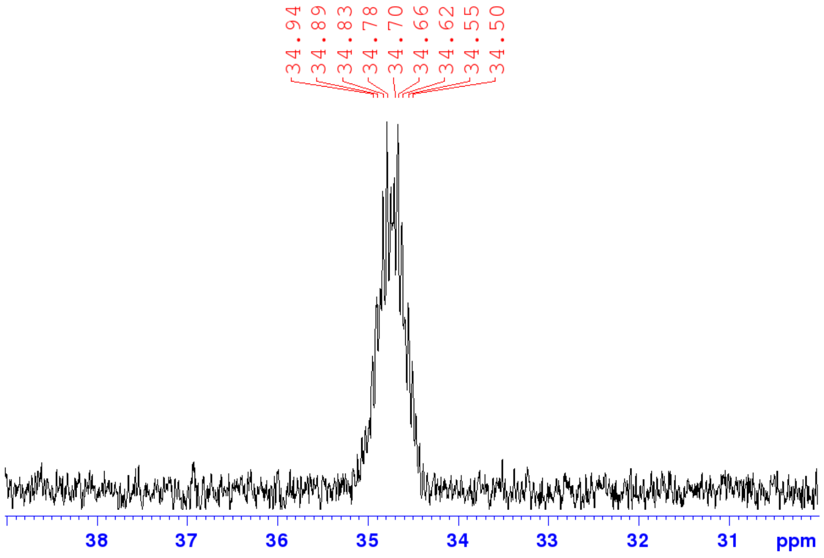

^{31}P NMR of **EP2** in CD_3OD

^{13}C NMR proton decoupled of **EP2** in CD_3OD

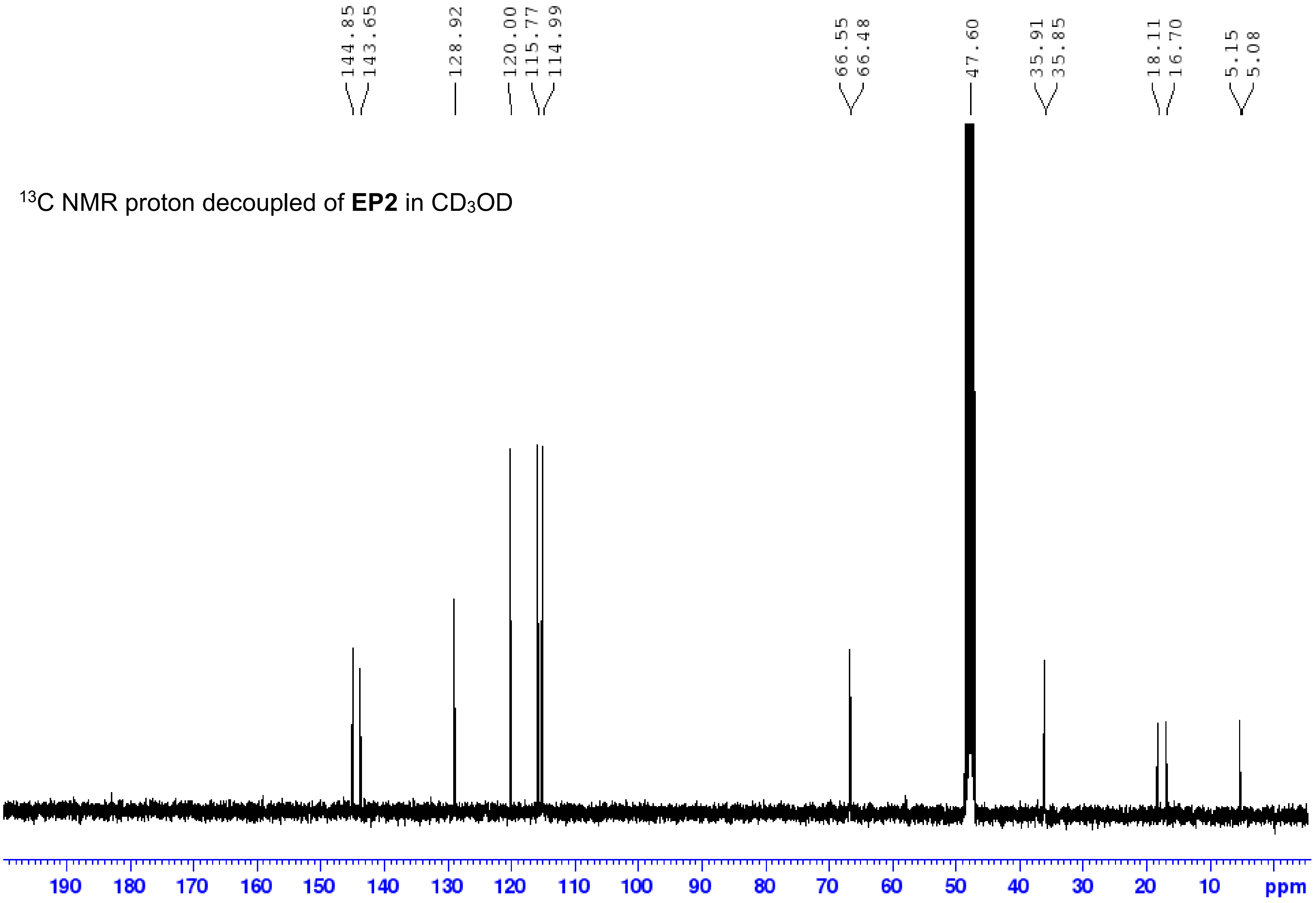

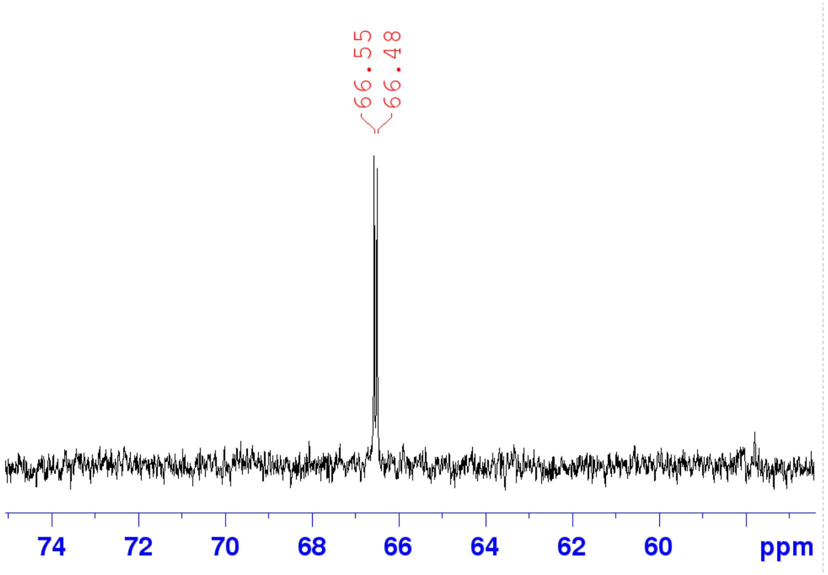

^{13}C NMR proton decoupled of **EP2** in CD_3OD: splitting pattern of C8 carbon.

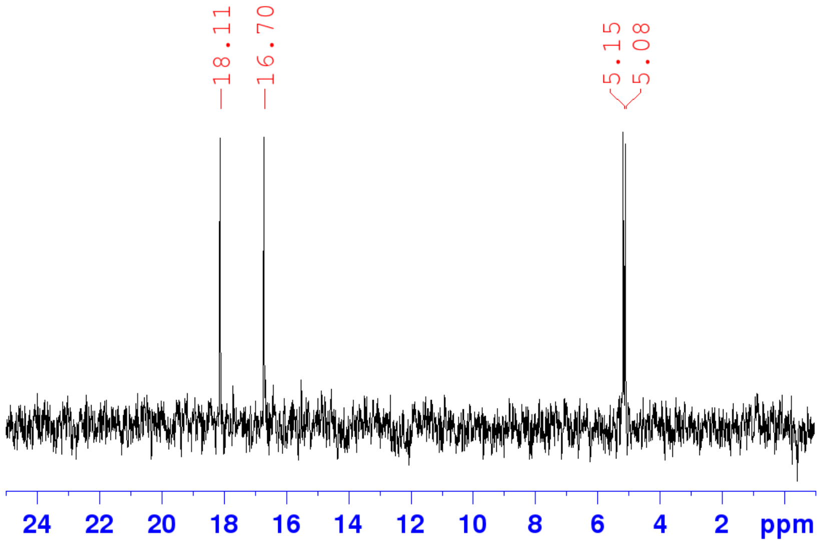

^{13}C NMR proton decoupled of **EP2** in CD_3OD: splitting pattern of $–CH_2CH_3$ carbons.

^{1}H NMR of **EP3** in CD_3OD

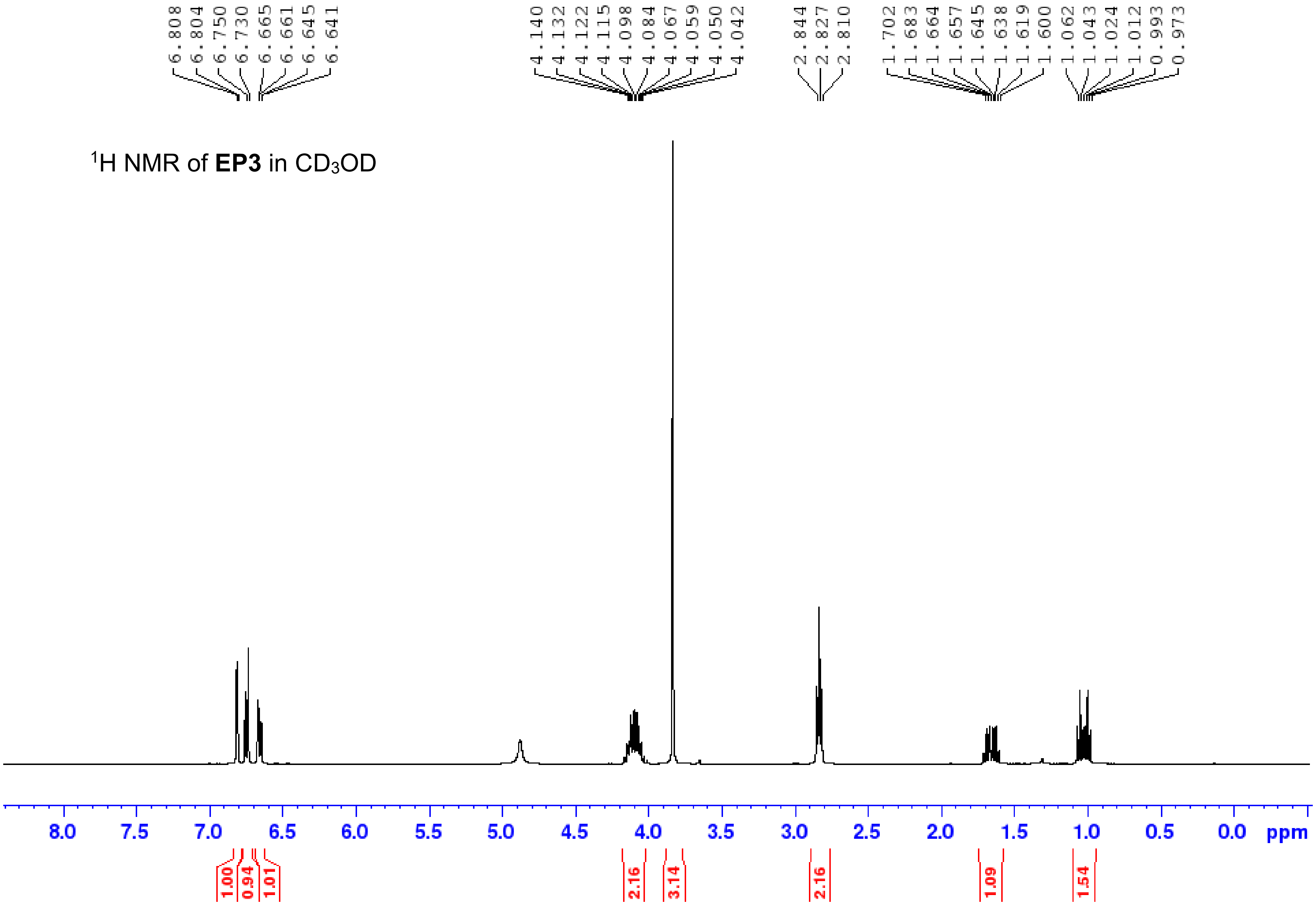

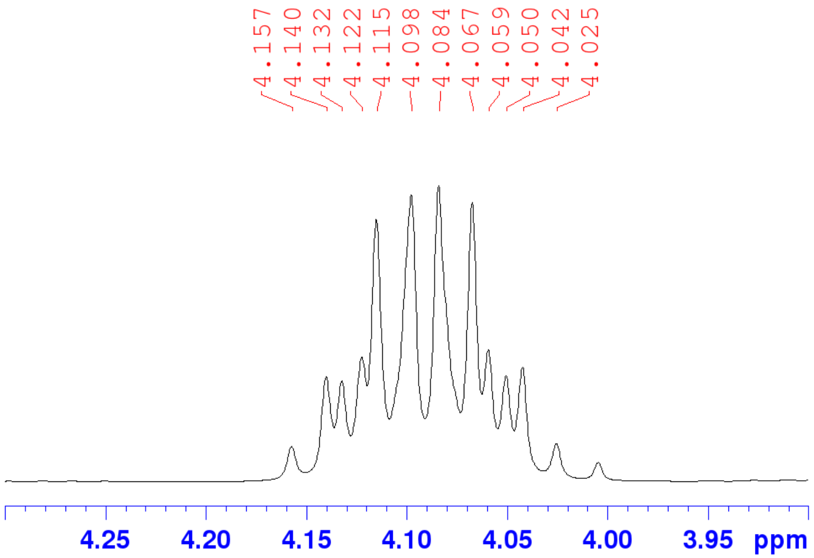

^{1}H NMR of **EP3** in CD_3OD: splitting pattern of H8 and H8' protons.

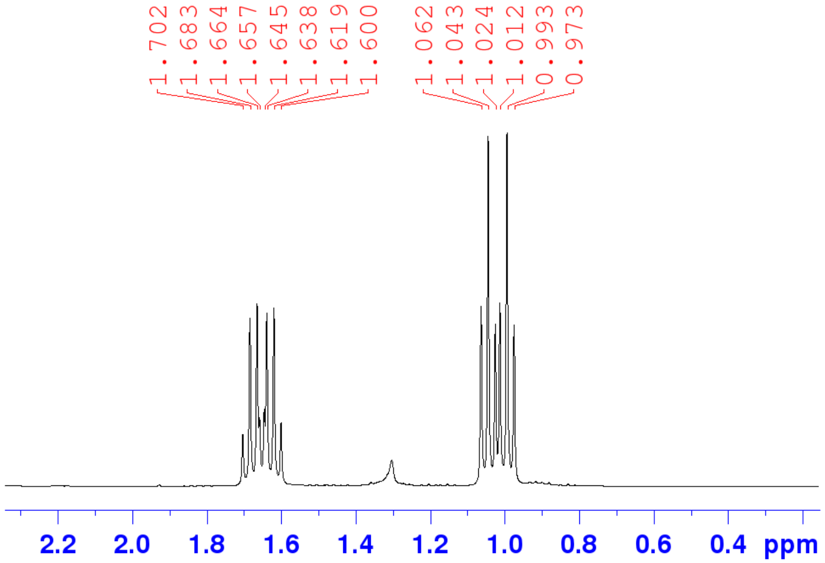

^{1}H NMR of **EP3** in CD_3OD: splitting pattern of $–CH_2CH_3$ ($AA'M_3X$) protons.

^{31}P NMR of **EP3** in CD_3OD

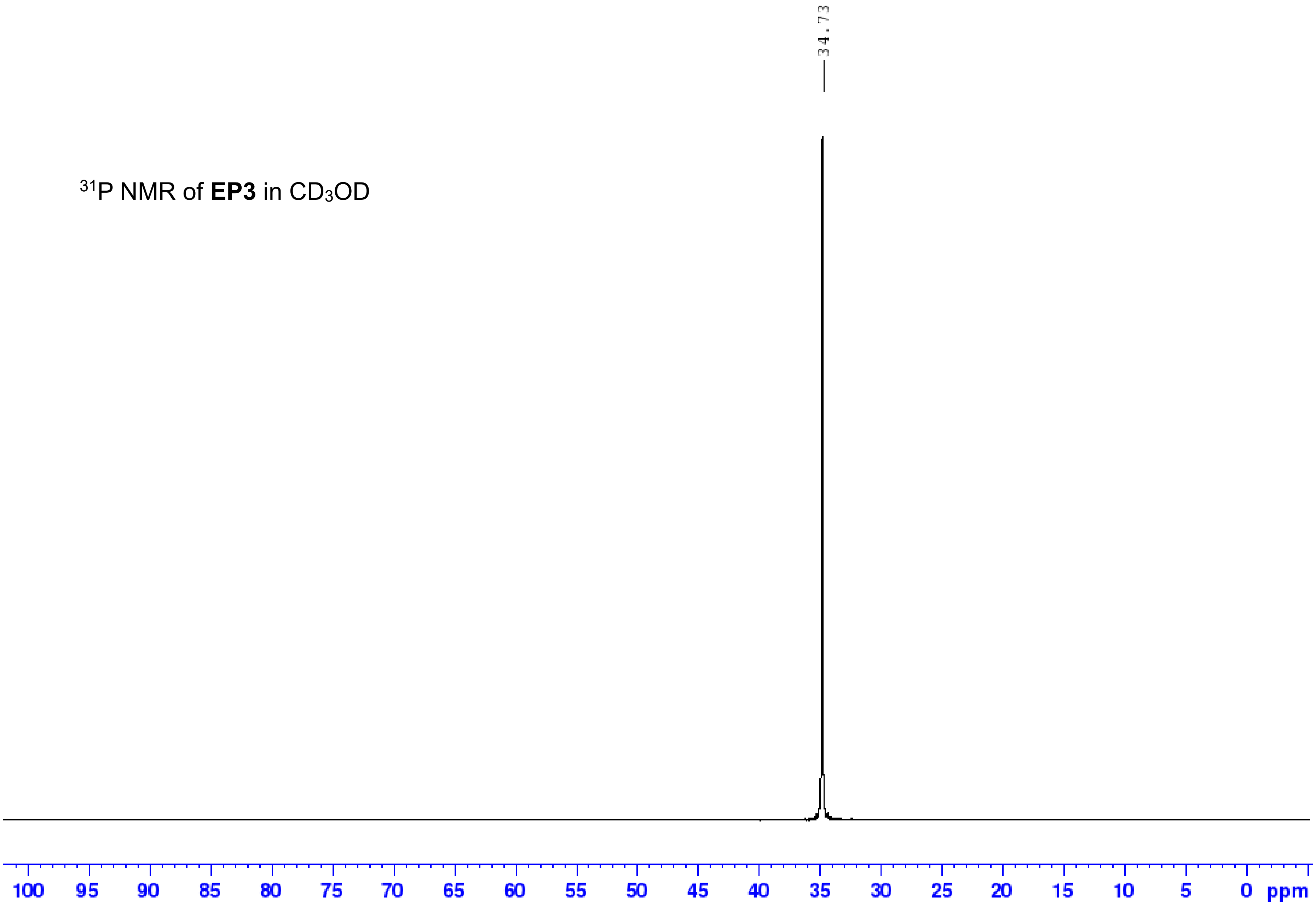

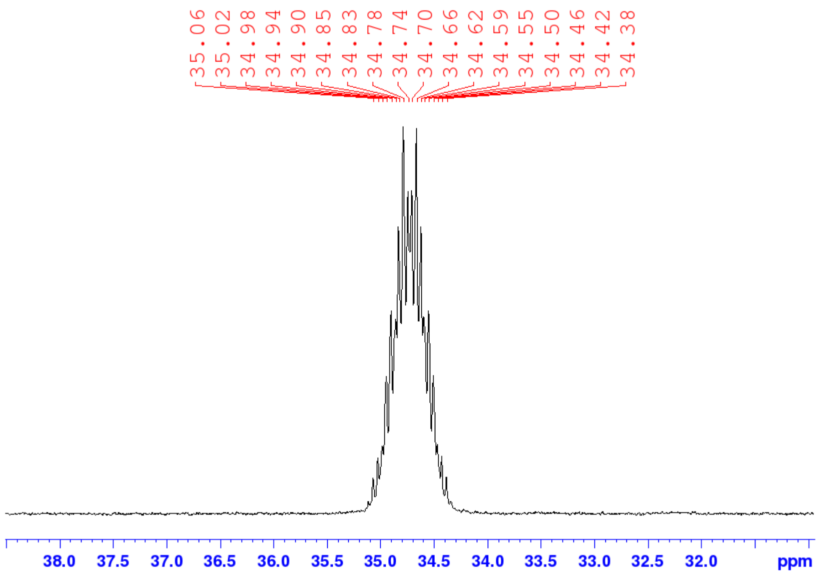

^{31}P NMR of **EP3** in CD_3OD

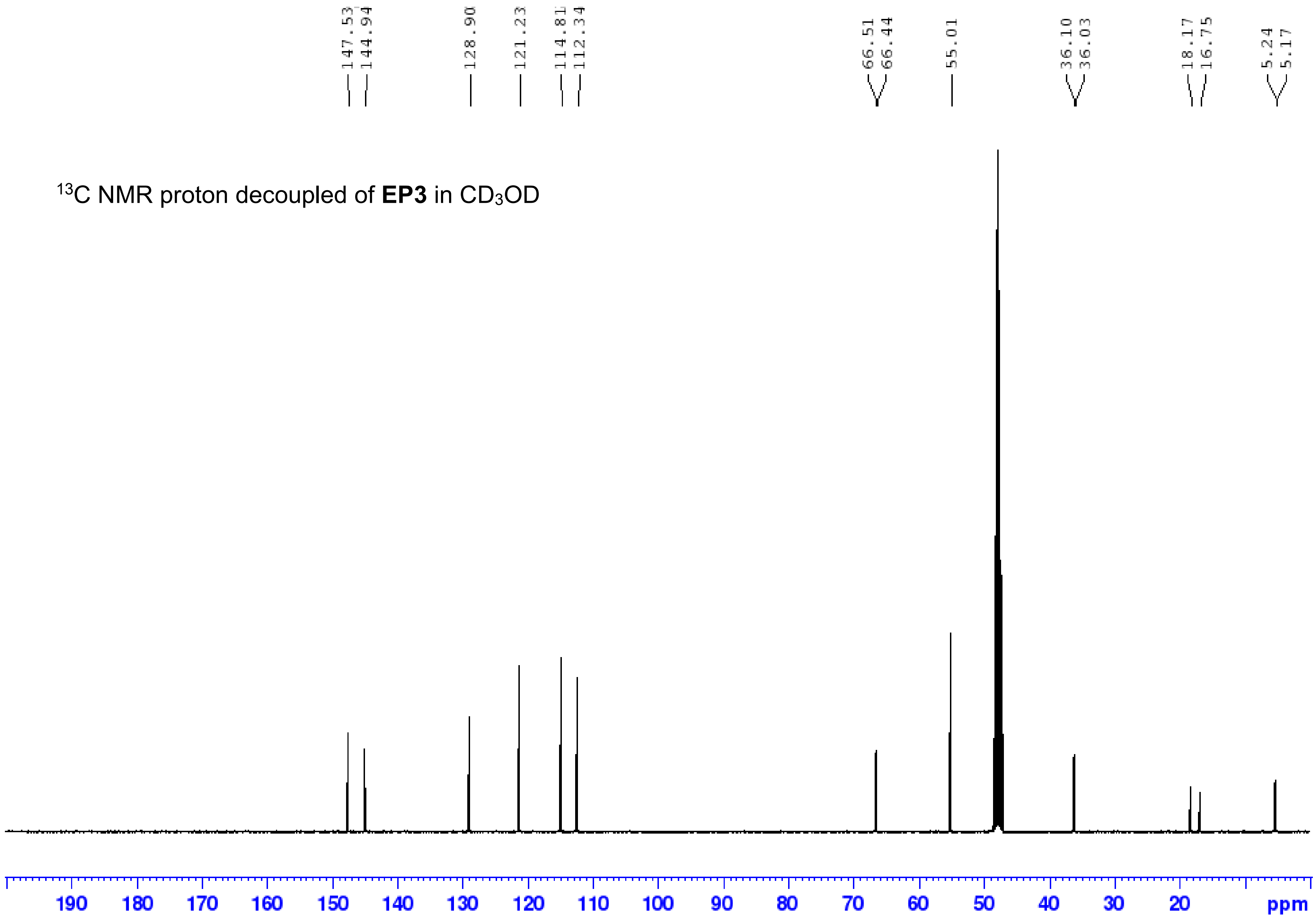

^{13}C NMR proton decoupled of **EP3** in CD_3OD

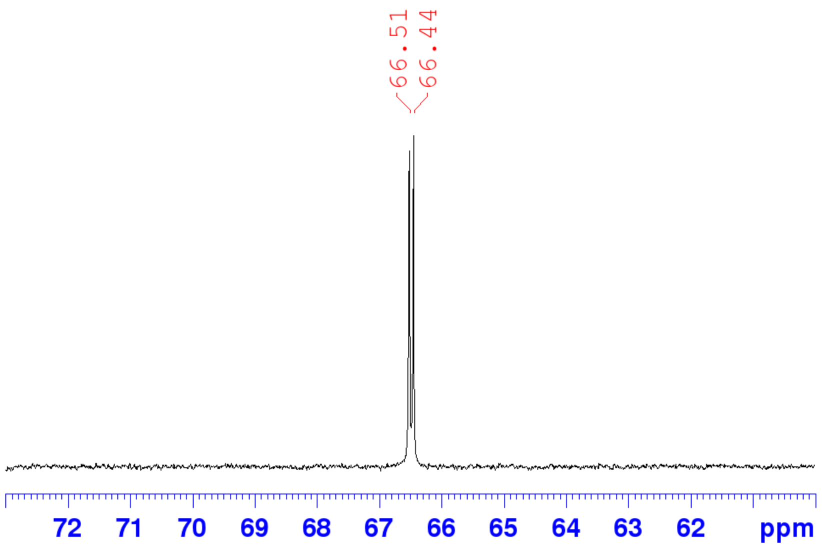

^{13}C NMR proton decoupled of **EP3** in CD_3OD: splitting pattern of C8 carbon.

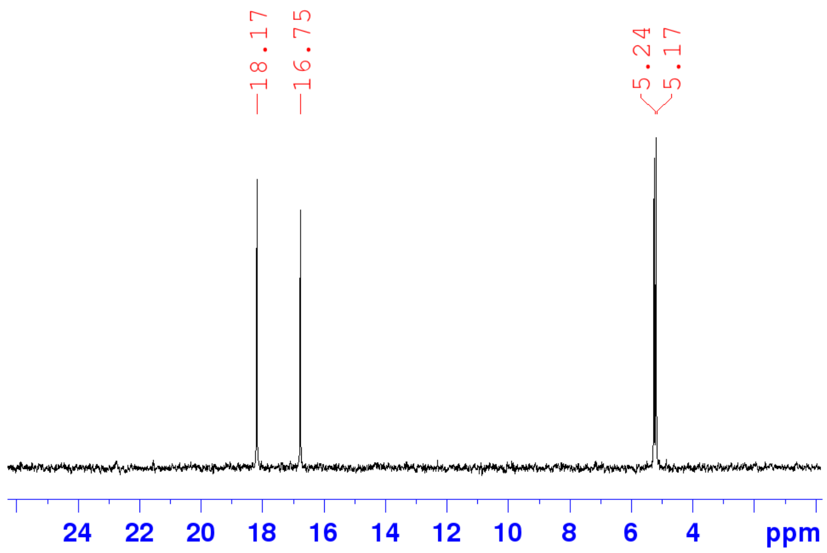

^{13}C NMR proton decoupled of **EP3** in CD_3OD: splitting pattern of $–CH_2CH_3$ carbons.

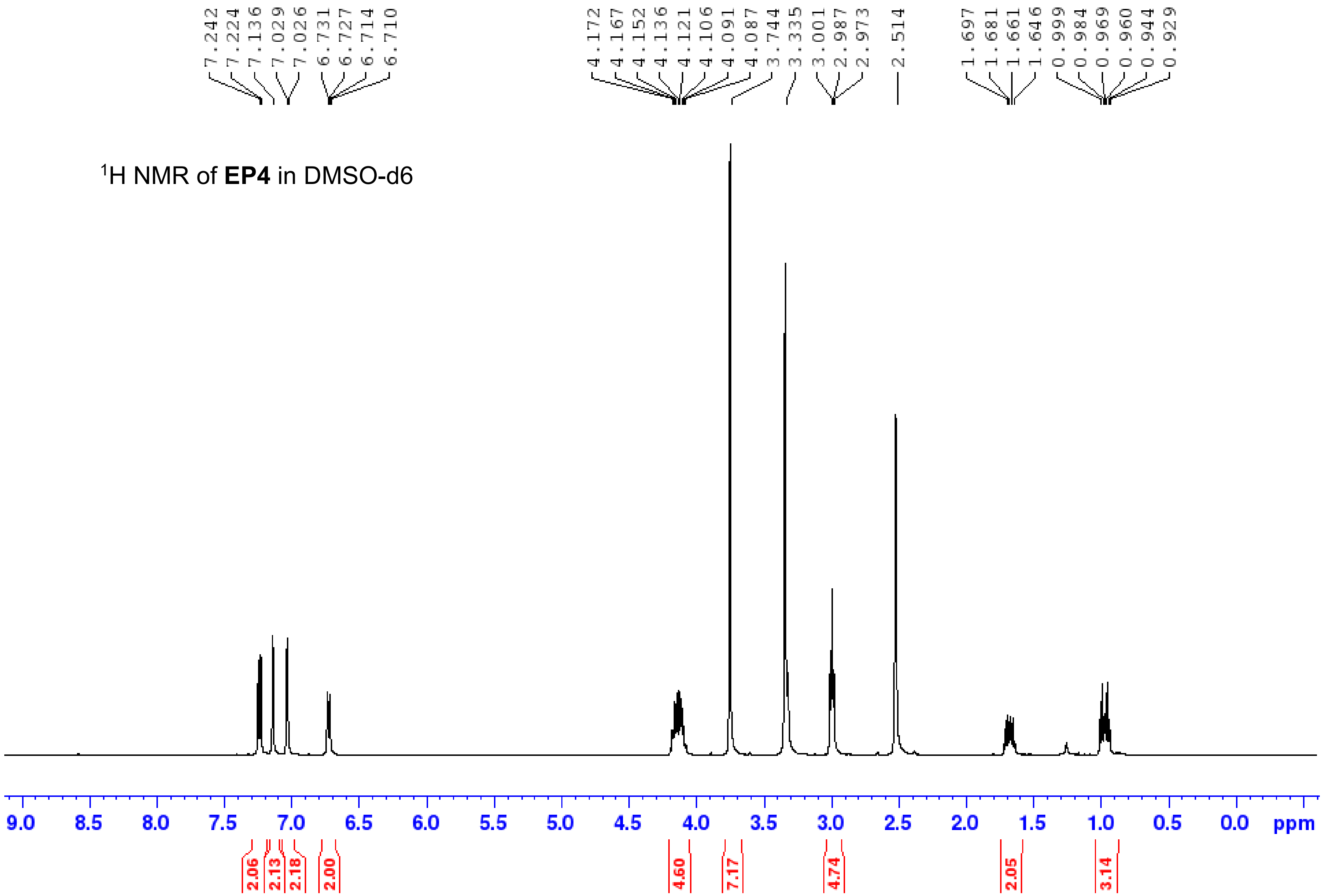

^{1}H NMR of **EP4** in DMSO-d6
7.242
7.224
7.136
7.029
7.026
6.731
6.727
6.714
6.710
4.172
4.167
4.152
4.136
4.121
4.106
4.091
4.087
3.744
3.335
3.001
2.987
2.973
2.514
1.697
1.681
1.661
1.646
0.999
0.984
0.969
0.960
0.944
0.929
9.0 8.5 8.0 7.5 7.0 6.5 6.0 5.5 5.0 4.5 4.0 3.5 3.0 2.5 2.0 1.5 1.0 0.5 0.0 ppm
2.06
2.13
2.18
2.00
4.60
7.17
4.74
2.05
3.14

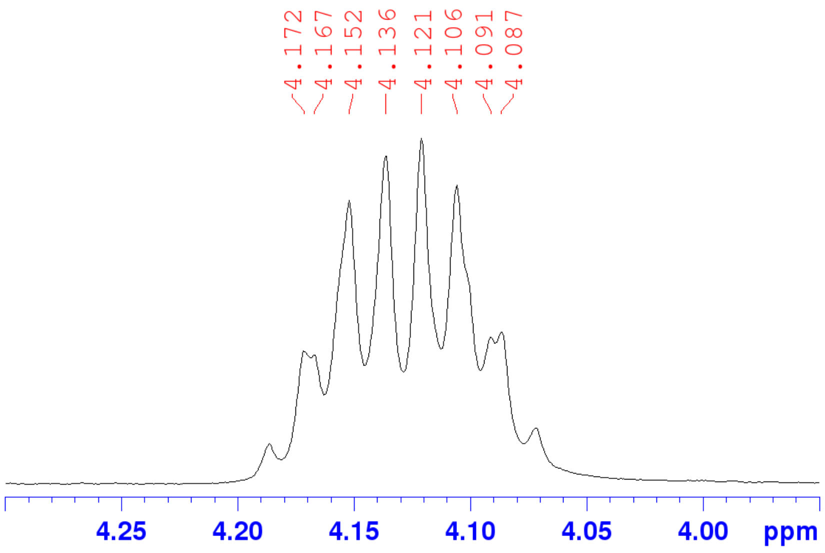

^{1}H NMR of **EP4** in DMSO-d6: splitting pattern of H8 and H8' protons.

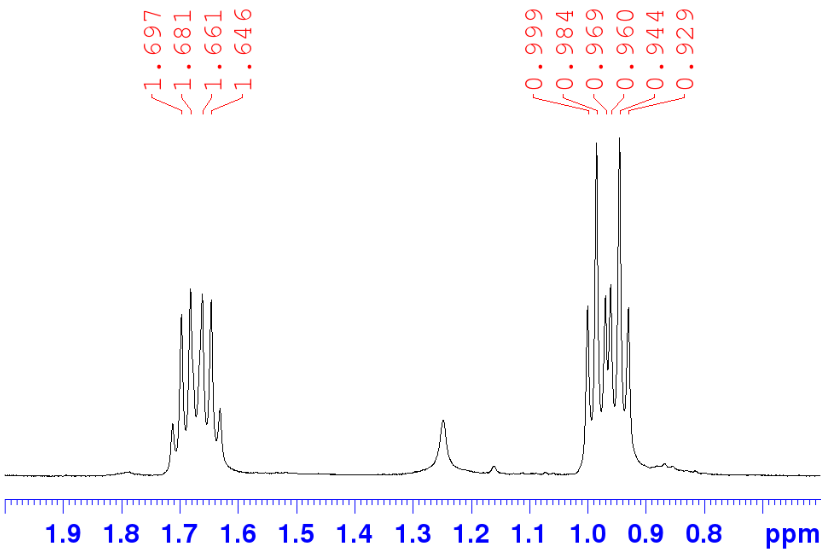

^{1}H NMR of **EP4** in DMSO-d6: splitting pattern of $–CH_2CH_3$ ($AA'M_3X$) protons.

^{31}P NMR proton decoupled of **EP4** in DMSO-d_6

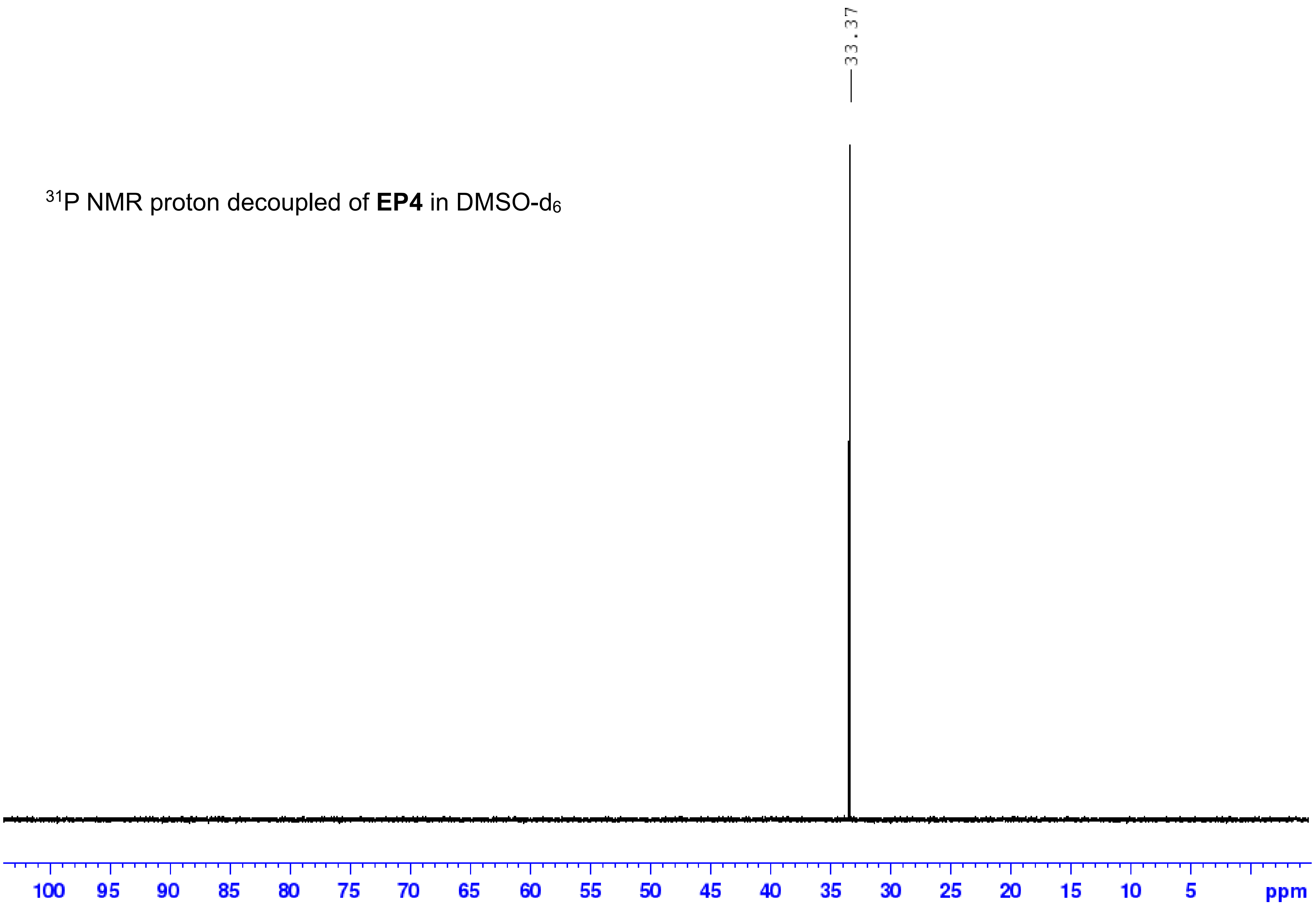

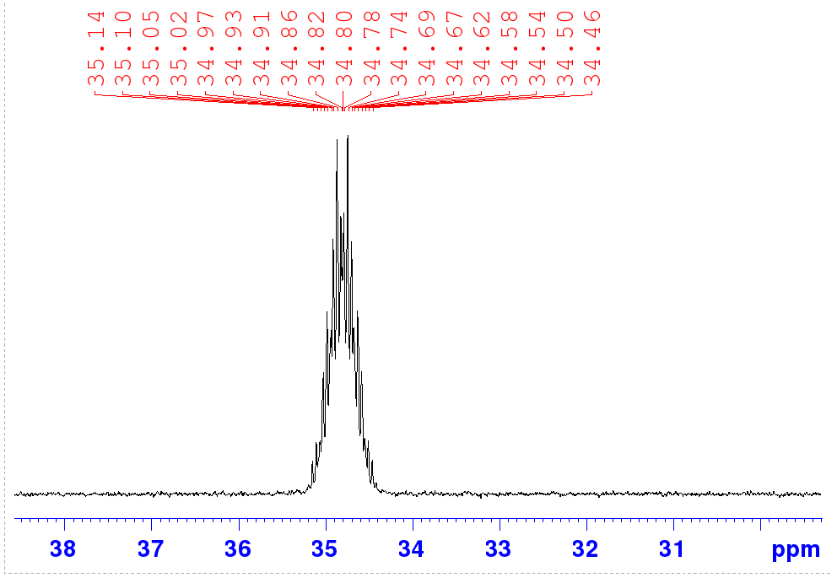

^{31}P NMR of **EP4** in DMSO-d_6

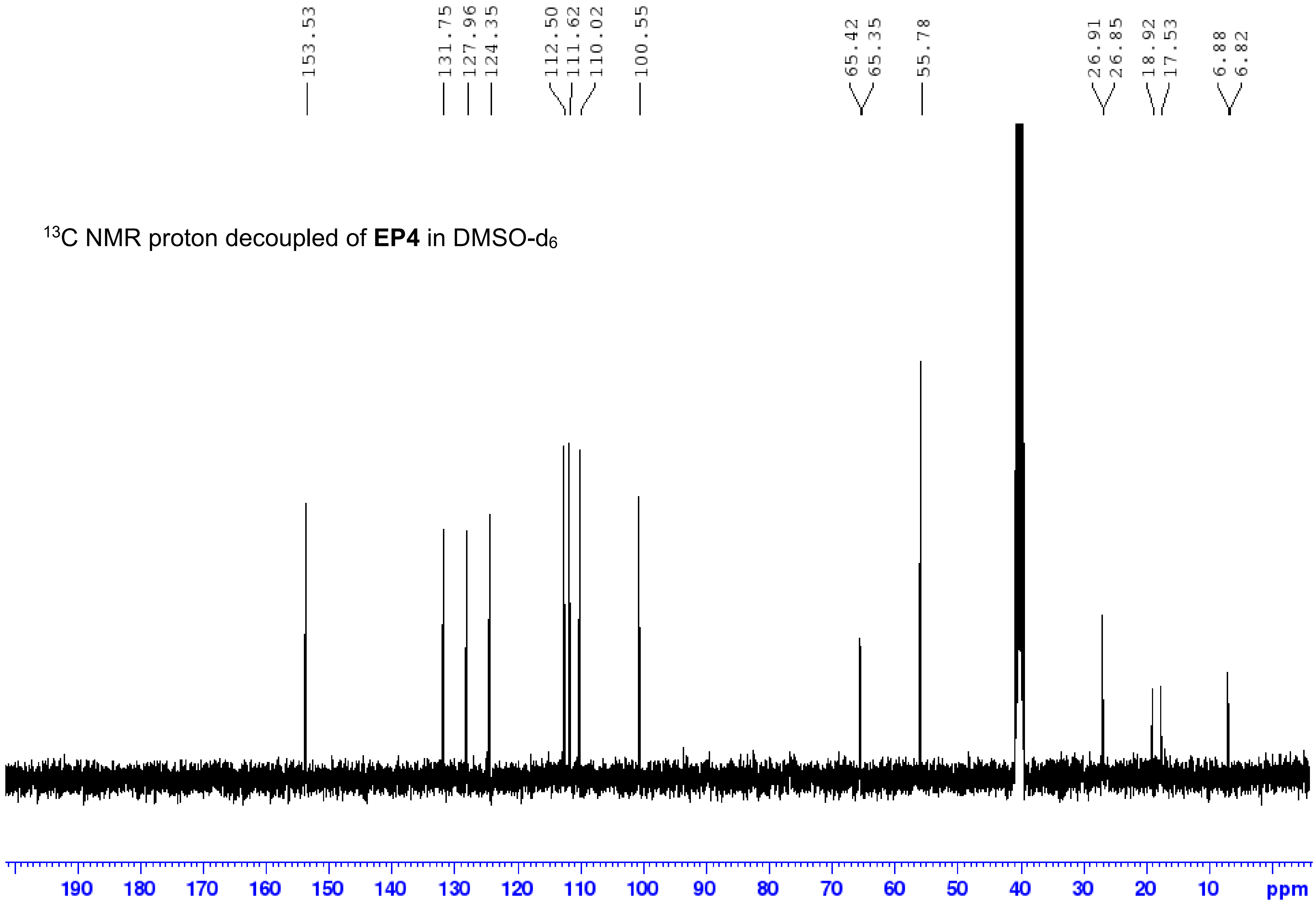

^{13}C NMR proton decoupled of **EP4** in DMSO-d_6

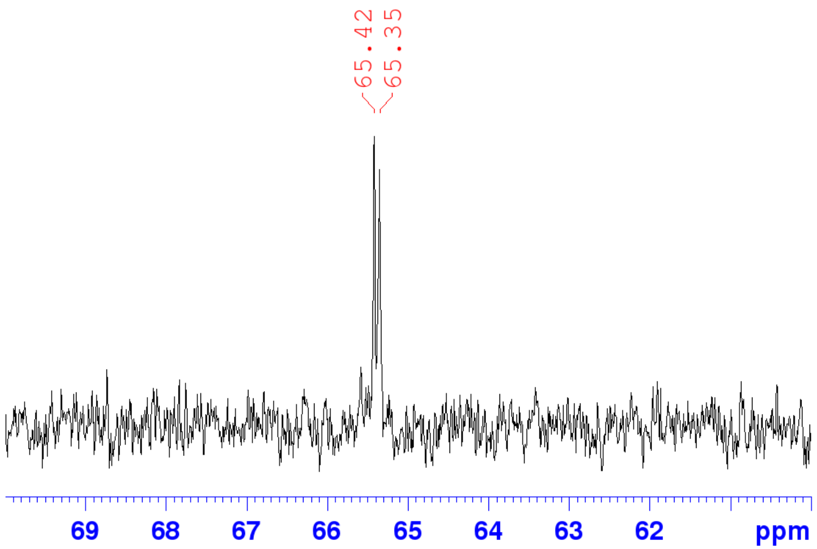

^{13}C NMR proton decoupled of **EP4** in DMSO-d_6: Splitting pattern of C8 carbon.

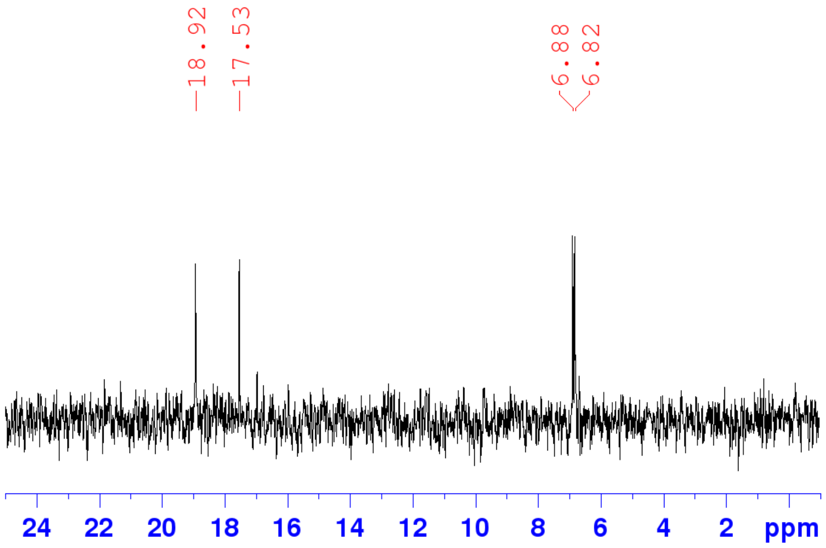

^{13}C NMR proton decoupled of **EP4** in DMSO-d_6: Splitting pattern of $–CH_2CH_3$ carbons.

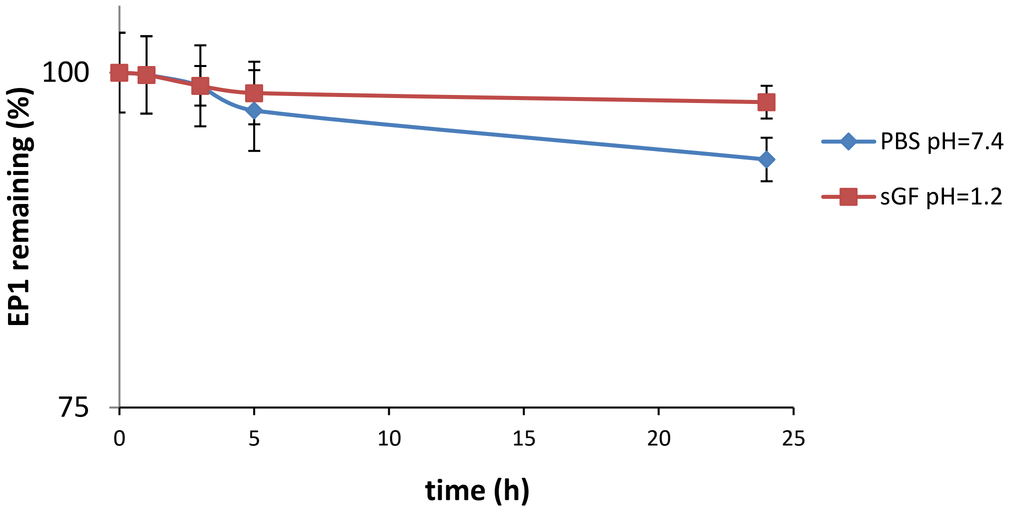

Figure S1. Stability of **EP1**: percentage of **EP1** remaining over time in PBS pH = 7.4 and sGF pH = 1.2 calculated by the integration of the corresponding HPLC peak. Bars are means ± SEM of three independent experiments (n = 3).

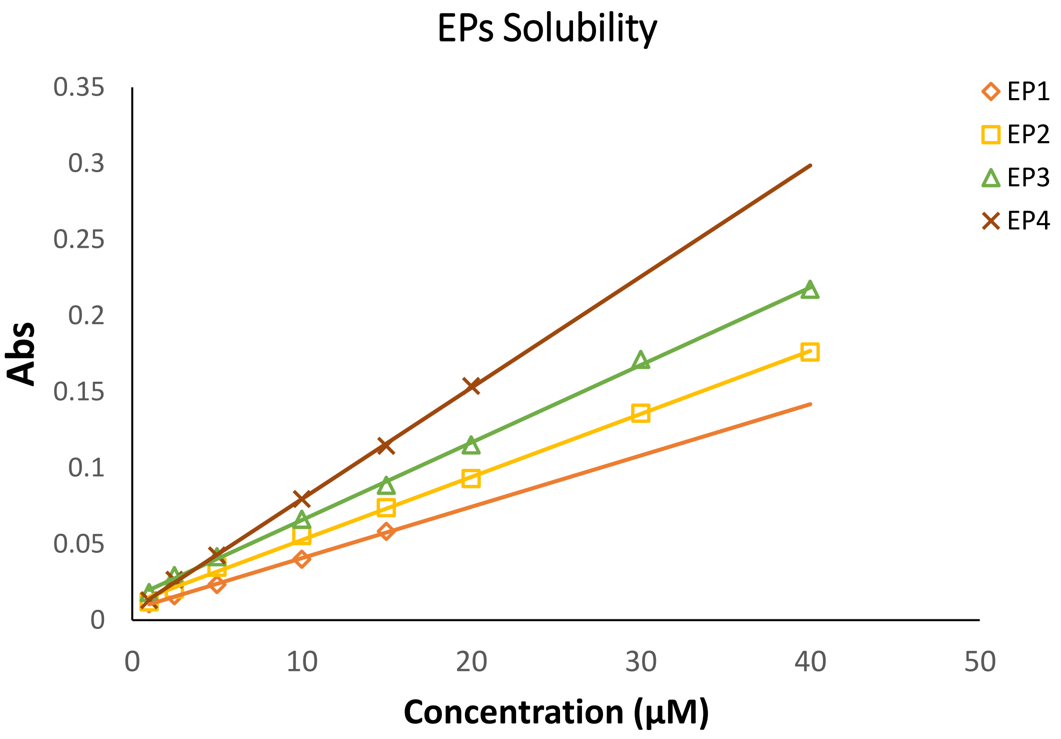

Figure S2. EPs water solubility in PBS buffer pH=7.4.

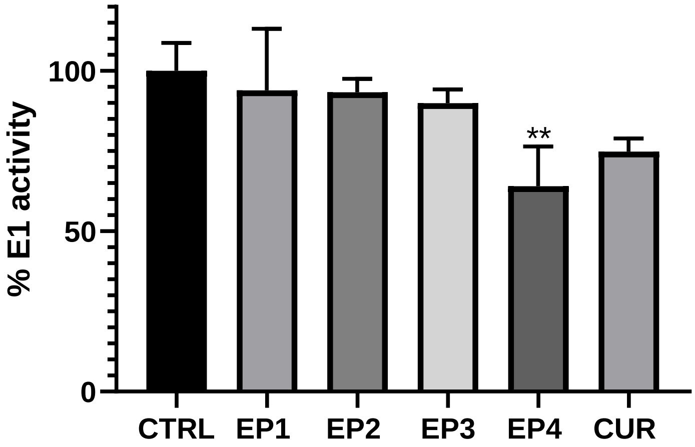

Figure S3. Pyrophosphate release during E1 enzyme activation in presence of **EP1-4** and curcumin. Ligand concentration was 5 µM.
